# The Calmodulin-like Calcium Binding Protein EhCaBP3 of *Entamoeba histolytica* Regulates Phagocytosis and Is Involved in Actin Dynamics

**DOI:** 10.1371/journal.ppat.1003055

**Published:** 2012-12-27

**Authors:** Saima Aslam, Sudha Bhattacharya, Alok Bhattacharya

**Affiliations:** 1 School of Life Sciences, Jawaharlal Nehru University, New Delhi, India; 2 School of Environmental Sciences, Jawaharlal Nehru University, New Delhi, India; Washington University School of Medicine, United States of America

## Abstract

Phagocytosis is required for proliferation and pathogenesis of *Entamoeba histolytica* and erythrophagocytosis is considered to be a marker of invasive amoebiasis. Ca^2+^ has been found to play a central role in the process of phagocytosis. However, the molecular mechanisms and the signalling mediated by Ca^2+^ still remain largely unknown. Here we show that Calmodulin-like calcium binding protein EhCaBP3 of *E. histolytica* is directly involved in disease pathomechanism by its capacity to participate in cytoskeleton dynamics and scission machinery during erythrophagocytosis. Using imaging techniques EhCaBP3 was found in phagocytic cups and newly formed phagosomes along with actin and myosin IB. *In vitro* studies confirmed that EhCaBP3 directly binds actin, and affected both its polymerization and bundling activity. Moreover, it also binds myosin 1B in the presence of Ca^2+^. In cells where EhCaBP3 expression was down regulated by antisense RNA, the level of RBC uptake was reduced, myosin IB was found to be absent at the site of pseudopod cup closure and the time taken for phagocytosis increased, suggesting that EhCaBP3 along with myosin 1B mediate the closure of phagocytic cups. Experiments with EhCaBP3 mutant defective in Ca^2+^ -binding showed that Ca^2+^ binding is required for phagosome formation. Liposome binding assay revealed that EhCaBP3 recruitment and enrichment to membrane is independent of any cellular protein as it binds directly to phosphatidylserine. Taken together, our results suggest a novel pathway mediating phagocytosis in *E. histolytica,* and an unusual mechanism of modulation of cytoskeleton dynamics by two calcium binding proteins, EhCaBP1 and EhCaBP3 with mostly non-overlapping functions.

## Author Summary

*Entamoeba histolytica* is one of the major causes of morbidity and mortality in developing countries. Phagocytosis plays an important role in both survival and virulence and has been used as a virulence marker. Inhibition of phagocytosis leads to a defect in cellular proliferation. Therefore, the molecules that participate in phagocytosis are good targets for developing new drugs. However, the molecular mechanism of the process is still largely unknown. Here, we demonstrate that Calmodulin-like calcium binding protein EhCaBP3 is involved in erythrophagocytosis. We show this by a number of different approaches including immunostaining of actin, myosin1B, EhCaBP1 and EhCaBP3 during uptake of RBC; over expression and down regulation of EhCaBP3, and over expression of calcium defective mutant of EhCaBP3. Our analysis suggests that EhCaBP3 can regulate actin dynamics. Along with actin and myosin 1B it can participate in both initiation and formation of phagosomes. The Ca^2+^-bound form of this protein is required only for progression from cups into early phagosomes but not for initiation. Our results demonstrate the complex role of Ca^2+^ binding proteins, EhCaBP1 and EhCaBP3 in regulation of phagocytosis in the protist parasite *E. histolytica* and the novel mechanisms of manipulating actin dynamics at multiple levels.

## Introduction

A variety of cell types, such as macrophages and neutrophils and many unicellular eukaryotes have the ability to engulf particles of size greater than 0.5 µm through a process called phagocytosis. In the former this process has evolved as one of the critical elements of host defence, while in the latter it serves as a mode of nutrition. *Entamoeba histolytica*, a parasite that colonizes the human gut and causes dysentery, is endemic in many developing countries and causes a high level of morbidity and mortality [1], [2]. Phagocytosis is considered to be important in *E. histolytica* pathogenesis, as a phagocytosis-deficient mutant showed reduced virulence [3]. In another study, the virulence potential of *E. histolytica* isolates could be directly correlated with their ability to phagocytose red blood cells (RBCs) [4].

Phagocytosis is initiated when a particle binds to a cell surface receptor, leading to local reorganization of actin cytoskeleton and providing the necessary force needed for the formation of phagocytic cups and phagosomes [5]–[7]. The rim of filamentous (F) actin (periphagosomal F-actin), surrounds early phagosomes and then progressively depolymerizes as the phagosome matures [5], [8], [9]. It is believed that this disassembly of the F-actin rim is necessary for phagosome maturation, as it may act as a barrier for phagosome-vesicle fusion [8]–[11]. Therefore, spatial and temporal regulation of actin dynamics is the key to controlling phagocytosis. This is achieved through a number of actin binding proteins (ABPs) [12]. ABPs are involved in regulating actin cytoskeleton dynamics at multiple levels; for example, promotion of nucleation and polymerization of F-actin by Arp2/3 complex and profilin [13], [14] and depolymerization of F-actin by ADF/cofilin and gelsolin [15]. Ca^2+^ is a prominent regulator that can exert multiple effects on structure and dynamics of actin cytoskeleton. Ca^2+^ transients during phagocytosis initiate these processes in many systems [16]–[18] including *E. histolytica* [19]. Cytoskeletal remodelling by Ca^2+^ may occur through Ca^2+^ binding proteins (CaBPs) that can sense alteration in Ca^2+^ concentration and undergo conformational change [20]–[22]. In *Dictyostelium discoideum*, a 34 kDa protein is involved in actin bundling in a calcium-regulated manner [23] and a 40 KDa protein restricts the length of actin filaments in the presence of Ca^2+^ [24], [25]. Ca^2+^ is also involved in other processes related to cytoskeleton remodeling, for example Ca^2+^-Calmodulin regulates actin polymerization via Fesselin [26] and a low molecular weight protein CBP1 in *D. discoideum* has been shown to regulate the reorganization of actin cytoskeleton during cell aggregation [27].

The role of actin in endocytic/phagocytic processes has been studied in different systems and cell types using a number of different inhibitors or pharmacological compounds [28]. Some of the results of these studies suggest that clathrin-coated vesicle formation may not require actin dynamics [29]. However, its role in post vesicle processing cannot be ruled out. In a different approach, over expression of Y282F/Y298F-FcgR, a signaling- dead mutant receptor in COS-7 cells is unable to signal to the actin cytoskeleton, but specifically binds IgG ligand, had no effect on phagocytosis [29]–[31]. In some of these cases it is thought that phagocytosis takes place via passive zipper mechanism in which ligand-receptor binding remains specific and strong but reversible due to the absence of actin polymerization. Passive engulfment is generally slower and produces much more variable phagocytic cups [32].

The genome analysis of *E. histolytica* has revealed 27 CaBPs with multiple EF-hand calcium binding domains [33]. Of these, EhCaBP1 has been studied in much more detail and it is now clear that EhCaBP1 is a central molecule involved in initiation of erythrophagocytosis along with EhC2PK, a C2 domain containing protein kinase [34]. EhC2PK accumulates at the site of RBC attachment in a Ca^2+^- dependent step and recruits EhCaBP1, which in turn brings actin filaments resulting in initiation of phagocytosis [34]. Ca^2+^ has been shown to participate in the initiation process at two levels. Firstly, it is necessary for membrane localization of EhC2PK and secondly, Ca^2+^-EhCaBP1 is required for phagocytic cups to progress towards phagosomes [34]. Therefore, Ca^2+^ has an important role in regulating erythrophagocytosis in *E. histolytica.*

A calmodulin-like calcium binding protein EhCaBP3 has been identified and partially characterized in *E. histolytica* [35]. Three dimensional structure, using nuclear magnetic resonance (NMR) spectroscopy, suggests that EhCaBP3 has a well folded N-terminal domain and an unstructured C-terminal counterpart, somewhat similar to calmodulin and EhCaBP1. Interestingly, EhCaBP3 was found in all three major cellular compartments; nucleus, cytoplasm and membrane [35]. In this report we show that EhCaBP3 is involved in the process of phagocytosis at both initiation and phagosome formation stages. *In vitro* experiments suggest that EhCaBP3 binds actin, and affects its polymerization and bundling. Therefore it is likely that EhCaBP3 regulates phagocytosis by participating in actin dynamics. Our studies also show that EhCaBP3 and EhCaBP1 have different roles though both are recruited early during phagocytosis. We conclude that *E. histolytica* displays unique mechanism of regulating phagocytosis using a number of novel calcium binding proteins not observed in any other system.

## Results

### Localization of EhCaBP3 during phagocytosis of RBCs

**Figure 1:**
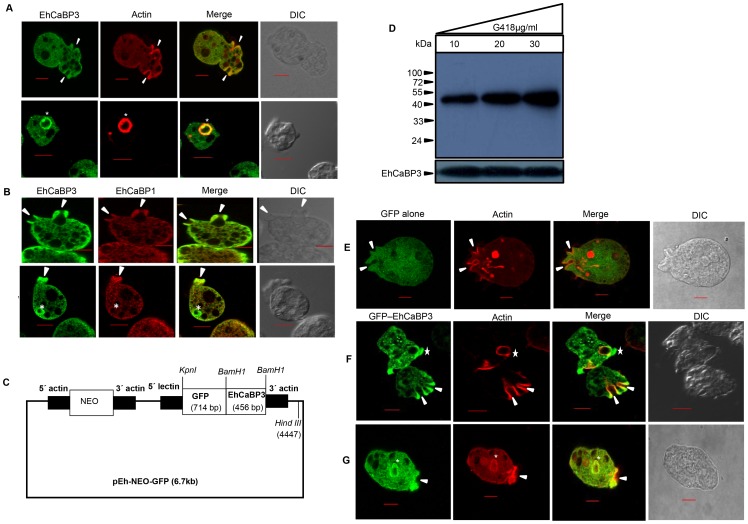
Distribution of EhCaBP3, EhCaBP1 and actin in *E. histolytica* during erythrophagocytosis. Localization of endogenous EhCaBP3 and actin (**A**) and EhCaBP3 and EhCaBP1 (**B**). Cells were grown for 48 h and incubated with RBC for 10 min at 37°C. The cells were then fixed and immunostained with rabbit anti- EhCaBP3 antibody followed by Alexa-488 (green) secondary antibody, and F-actin was stained with TRITC-phalloidin (red) and viewed using confocal microscope. The secondary antibody used for EhCaBP1 was Alexa-556 (red). Arrow heads represent phagocytic cups and an asterisk mark showing the enrichment of EhCaBP3 in phagosome. Bar represents 10 µm. (DIC, differential interference contrast). (**C**) Schematic representation of GFP-EhCaBP3 constructs. (**D**) Western blot of amoebic cell lysates expressing GFP-EhCaBP3 construct at different G418 concentrations (10, 20, 30 µg ml^−1^). Thirty microgram of the lysate was loaded in each lane and the blot was probed with anti-GFP antibody. The blots were stripped and re-probed with anti-EhCaBP3 antibody. The EhCaBP3 antibody stains both endogenous EhCaBP3 band at 17 kDa and GFP-fused EhCaBP3 at 43 kDa. (**E–G**) Immunolocalization of GFP-EhCaBP3 in *E.histolytica* during erythrophagocytosis. Cells expressing GFP alone (**E**) or GFP-EhCaBP3 (**F, G**) were incubated with RBC, followed by fixation and immunostaining. GFP− tagged protein were labeled using anti-GFP antibody and F-actin by phalloidin (red), followed by Alexa 488 (green) secondary antibody. (Scale bar, 10 µm; DIC, differential interference contrast).

Ca^2+^ is required for phagocytosis in *E. histolytica* as chelation of cytoplasmic Ca^2+^ blocks phagocytosis [19]. Therefore, it is expected that CaBPs may be participating in phagocytosis as Ca^2+^ sensors. We have earlier shown the involvement of one of the calcium sensing CaBPs of *E. histolytica*, EhCaBP1 in erythrophagocytosis [19], [21]. EhCaBP3 was identified as a calmodulin-like calcium binding protein of *E. histolytica* as its structure showed similarity with calmodulin [35]. Since multiple CaBPs are likely to be involved in different steps of phagocytosis, the subcellular localization of EhCaBP3 was checked during RBC uptake by immunostaining with specific anti-EhCaBP3 antibody. The results are shown in **Figure 1** **.** Fluorescence signals clearly showed that EhCaBP3 was present in phagocytic cups, as has been shown for EhCaBP1 [19]. Actin was also observed to line the cups and the complete superimposition of both EhCaBP3 and actin suggested that both proteins are colocalized at the phagocytic cups (**Figure 1A**). EhCaBP3 was also found on early phagosomes along with actin. Superimposition of both molecules suggested that both EhCaBP3 and actin are also co-localized at the newly formed phagosomes.

Our earlier studies had shown that EhCaBP1 was found only at cups and not on phagosomes. Therefore relative localization of EhCaBP1 and EhCaBP3 were studied in actively phagocytosing cells in order to see functional differences between the two CaBPs, using antibodies against EhCaBP1 (red) and EhCaBP3 (green). Since we wanted to see both phagocytic cups and phagosomes, amoebic cells were incubated with RBCs for different times. As expected EhCaBP1 was observed only in the phagocytic cups whereas EhCaBP3 was found in both phagocytic cups as well as in early phagosomes (Figure 1B). The results suggest that EhCaBP3 is likely to be involved in erythrophagocytosis and it may be functionally different from EhCaBP1.

In order to check if EhCaBP3 may also participate in phagocytosis of other particles, EhCaBP3 was immunostained during phagocytosis of CHO cells and the results are shown in Figure S1. Fluorescent signals were found in the cups that are in the process of phagocytosing CHO cells. However, it was not clear whether any significant signal was present around the phagosomes, as observed with RBCs (compare Figure S1 and Figure 1). Phagosome with low intensity staining could be discerned in some cases and these are marked with asterisk. Many CHO cells formed tunnel like structure during phagocytosis and EhCaBP3 was localized at the tip (marked by an arrow). These tunnel-like structures have also been observed before [36]. The results suggest that EhCaBP3 may also be involved in phagocytosis of CHO cells. However, the extent of participation and the exact roles may be different from that of RBCs. We have further characterized the role of EhCaBP3 in phagocytosis using RBC uptake as our model.

### Dynamics of GFP-tagged EhCaBP3 during erythrophagocytosis

Dynamics of EhCaBP3 recruitment and release during erythrophagocytosis was studied by expressing EhCaBP3 in *E. histolytica* cells as a GFP fusion protein on a plasmid vector maintained in the presence of G418 (Figure 1C). While there was no change in the expression of endogenous EhCaBP3 (17 kDa), the expression of GFP-EhCaBP3 (43 kDa) increased with increasing concentration of G418 as seen by western blotting using anti-GFP antibodies which do not stain endogenous EhCaBP3 (Figure 1D). There was no change in the levels of endogenous EhCaBP3 visualized by anti-EhCaBP3 antibody under the same conditions. Since it is likely that GFP tagged proteins may not behave like native proteins we checked the localization of GFP-EhCaBP3 during erythrophagocytosis using anti-GFP antibodies. Confocal microscopy revealed that GFP-tagged EhCaBP3 (but not GFP alone) enriched at phagocytic cups and early phagosomes along with actin (Figure 1E, 1F, 1G), suggesting that GFP-EhCaBP3 behaves in a similar way as endogenous EhCaBP3.

**Figure 2:**
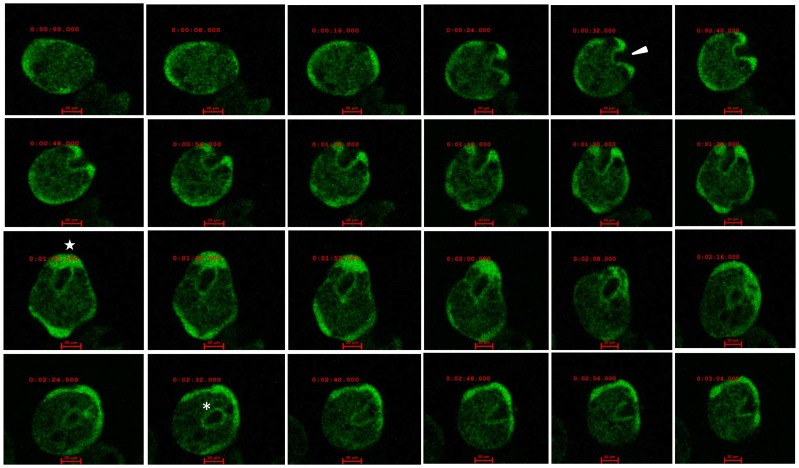
Time lapse imaging of GFP-EhCaBP3. Micrograph showing the de novo formation of a phagocytic cup during phagocytosis of RBC by an amoeba expressing GFP-EhCaBP3. The montage shows a time series of a representative cell showing the formation of phagocytic cup (arrow head), the closure of cup (star) and finally a complete phagosome (asterisk). Bar represents 20 µm.

The results reported so far show that EhCaBP3 is required both at the initiation and end stages of phagocytosis. It appears to redistribute during the whole process. In order to observe this dynamic behaviour of EhCaBP3, time-lapse fluorescence microscopy was used with cells expressing GFP-tagged molecules in the presence of RBCs. The results clearly showed that EhCaBP3 first accumulated rapidly at the site of RBC attachment before moving towards the tip of the cups (**Figure 2**). EhCaBP3 was present at the time of scission and remained even after complete phagosomes were formed and detached from the membrane. The whole process took about 3 min after addition of RBCs (**supplementary movie S1**).

### Interaction of EhCaBP3 with actin

**Figure 3:**
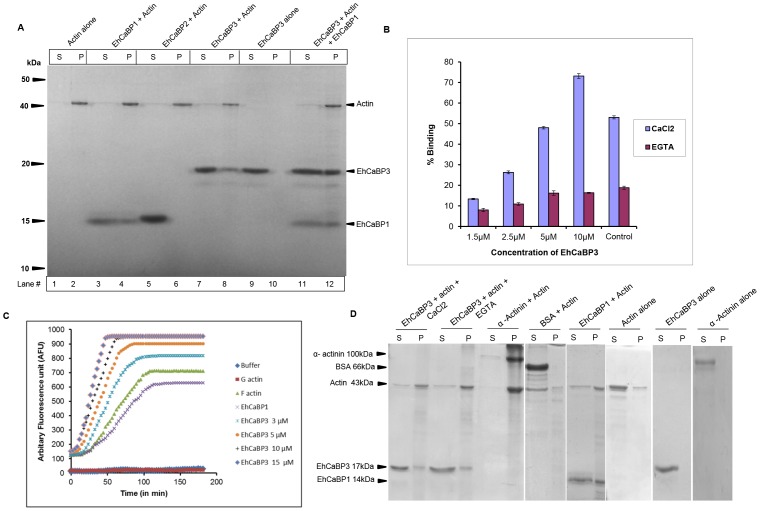
Binding of EhCaBP3 to actin. (**A**) Co-sedimentation assay of EhCaBP3 with F-actin. Polymerized F-actin (5 µM) was incubated with purified recombinant proteins EhCaBP3 (5 µM, lanes 7, 8), EhCaBP1 (5 µM, lanes 3, 4) and EhCaBP2 (5 µM, lanes 5, 6) as indicated. EhCaBP3 and EhCaBP1 were also incubated with actin (lanes 11, 12). EhCaBP3 alone was incubated without actin (lanes 9, 10). Actin alone without CaBP3 (lanes 1, 2). Ultra-centrifugation (100,000×g) was performed to separate supernatant (S) and pellet (P) fractions, followed by SDS-PAGE. (**B**) EhCaBP3 binding to G-actin. 5 µM Actin was coated overnight on 96well plate at 4°C, followed by blocking with BSA. EhCaBP3 was added in increasing concentrations as indicated in the presence (5 mM CaCl_2_) or the absence of Ca^2+^ (2 mM EGTA) and EhCaBP1 (5 µM) was used as a control. This was followed by incubation with anti-EhCaBP3 or anti-EhCaBP1 antibodies. The amount of bound protein was detected using anti-rabbit IgG-HRPO. Absorbance was determined at 405 nm. The histogram shows relative mean intensity ± SD of three independent experiments. (**C**) Effect of EhCaBP3 on actin polymerization. Pyrene-labeled actin was polymerized without and with indicated concentrations of EhCaBP3 as described in “Materials and methods”. The fluorescence of pyrene was observed at 407 nm. (**D**) F-actin bundling in presence of EhCaBP3. Polymerized F-actin (3 µM) was incubated with EhCaBP3 (5 µM) in presence of 5 mM CaCl_2_ and 2 mM EGTA for 2 h. The samples were then centrifuged at (10,000×g) for 15 min. Under this condition, unbundled F-actin remains in supernatant and bundled F-actin sediments. Both supernatant (S) and pellet (P) were analyzed by SDS-PAGE.

The results shown earlier clearly indicate colocalization of EhCaBP3 with F-actin in the context of phagocytosis. This may be brought about by binding of EhCaBP3 to F-actin directly or indirectly through a third molecule. In order to check these possibilities a direct binding assay of EhCaBP3 to F-actin was carried out by co-sedimentation. Polymerized actin was incubated with recombinant purified EhCaBP3 or other indicated proteins, and the complex was centrifuged and analysed by SDS-PAGE. Actin alone was found in the pellet fraction suggesting that the preparation contained mainly polymerized or F-actin. In the absence of actin, the pellet did not contain EhCaBP3 (Figure 3A, lane10). However, when actin was present EhCaBP3 was found in the pellet fraction (Figure 3A, lane 8). EhCaBP1 was also present along with polymerized actin in the pellet, as expected, being an actin-binding protein (Figure 3A, lane 4). In contrast, EhCaBP2, a close homolog of EhCaBP1 with a different function did not co-sediment with F-actin [21] (Figure 3A, lane 6). When actin was incubated with both EhCaBP1 and EhCaBP3, interestingly a complex containing both CaBPs and actin was detected in the pellet (Figure 3A, lane12). This could be due to a ternary complex (Actin, EhCaBP1 and EhCaBP3) or two separate binary complexes (Actin and EhCaBP3; Actin and EhCaBP1), which cannot be distinguished at present. Our results suggest that EhCaBP3 can bind F-actin directly.

To test the binding of EhCaBP3 to G-actin, a solid-phase assay was performed in the presence or the absence of Ca^2+^. It was observed that EhCaBP3 bound G-actin in the presence of Ca^2+^. However, the binding was inhibited by 75% when EGTA was added (Figure 3B). This data suggests that EhCaBP3-G-actin interaction requires Ca^2+^.

We then checked if binding of EhCaBP3 affects properties of actin, as EhCaBP1 was shown to alter the bundling of actin but not its polymerization [21]. EhCaBP1 was used as a negative control as it does not have any effect on actin polymerization [21]. First we tested if EhCaBP3 has an effect on actin polymerization by using pyrene-labelled G-actin. The rate of actin polymerization increased on adding increasing amount of EhCaBP3 reaching a saturation at about 10 µM. At this concentration both the rate as well as the value at saturation was higher by 50% compared to the control. No change in the rate of polymerization was observed in the presence of EhCaBP1 as expected (Figure 3C). To test whether EhCaBP3 influences bundling property of actin, the assay was performed in the presence and the absence of Ca^2+^. Majority (91%) of the actin was found in the supernatant fraction when actin alone, or in the presence of BSA were incubated without EhCaBP3, suggesting that there was no significant amount of actin in the form of bundles (Figure 3D). However, incubation of actin with EhCaBP3 led to bundling of actin as the majority of actin was in the pellet fraction. The result with EhCaBP3 was similar to that with known actin bundling agents, such as EhCaBP1 and alpha actinin [21]. In both cases actin was recovered from the pellet fraction after incubation. Our data also shows that actin bundling property of EhCaBP3 is independent of Ca^2+^ as actin was seen in the pellet in the presence and the absence of Ca^2+^. Our results suggest that EhCaBP3 is an actin remodelling protein and that EhCaBP1 and EhCaBP3 have different functional effects on actin.

### Identification of Myosin 1B as an interacting partner of CaBP3

**Figure 4:**
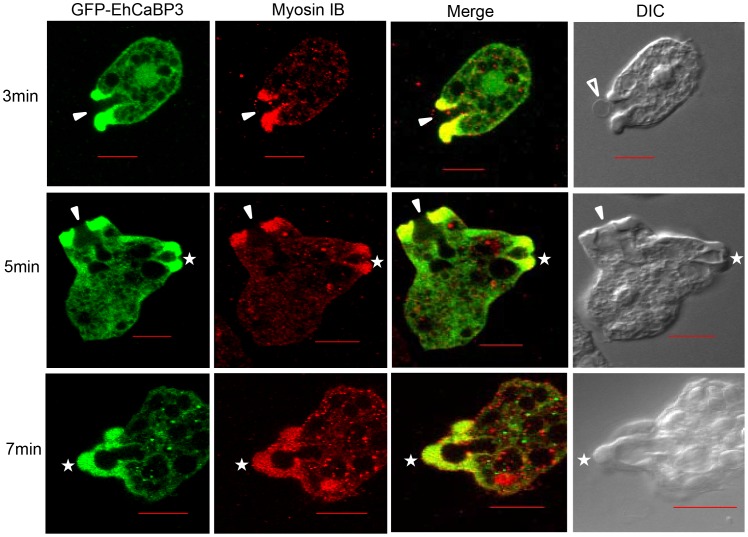
Immunolocalization of EhCaBP3 and myosin IB in erythrophagocytosing *E. histolytica.* Cells were first grown for 48 hours and then incubated with RBCs for indicated time points at 37°C. These were then fixed, permeabilized followed by immunostaining with anti-GFP and anti-myosin IB antibodies, followed by Alexa 488 (green, EhCaBP3) and Alexa 556 (red, myosin IB) secondary antibodies. Scale bar: 10 µm. Solid arrow indicates the region of enriched co-localization of these two proteins at phagocytic cups, open arrow head denotes attached RBC and star shows their co-localization during closure of phagocytic cups.

**Figure 5:**
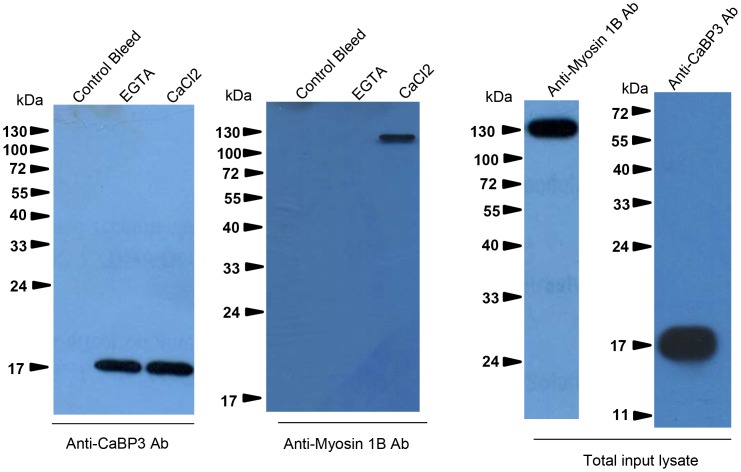
Interaction of EhCaBP3 with myosin 1B. Total *E. histolytica* lysate (800 µg) was incubated with Sepharose-anti-CaBP3 antibody conjugate for 6 h at 4°C with shaking. The beads were then washed and the bound material was then eluted and analysed by western blotting and immunostained with anti-myosin 1B antibody raised in rabbit. The blot was reprobed with anti-CaBP3 antibody raised in mice. The total input lysate was also probed for the presence of EhCaBP3 and myosin 1B by their respective antibodies.

Myosin IB is thought to be one of the proteins that interact with actin and is involved during some of the cellular processes in *E. histolytica*, such as phagocytosis [37]. The relationship between EhCaBP3 and myosin IB was investigated in the context of erythrophagocyosis using GFP-EhCaBP3 expressing cells and anti-myosin IB antibodies. *E. histolytica* cells were incubated with RBCs for different time points so as to capture different stages of phagocytosis. In all stages, that is, from cups to newly formed phagosomes, GFP-EhCaBP3 and myosin IB were found to co-localize (Figure 4). The presence of both myosin IB and EhCaBP3 at the tip just before phagosome closure (denoted by star) suggests that EhCaBP3 along with myosin IB may be involved in the process of phagosome closure. Localization of EhCaBP3 with myosin 1B suggests that these proteins might interact with one another. To confirm this, co-immunoprecipitation was carried out using immobilized anti-EhCaBP3 antibody and total cell lysate of *E. histolytica* trophozoites. The result is shown in Figure 5. While anti-EhCaBP3 antibody precipitated myosin 1B along with EhCaBP3 in the presence of Ca^2+^ (Figure 5), no myosin 1B was observed when EGTA was added, suggesting that Ca^2+^ is essential for their interaction.

### Role of Ca^2+^ in EhCaBP3-actin interaction and phagocytosis

**Figure 6:**
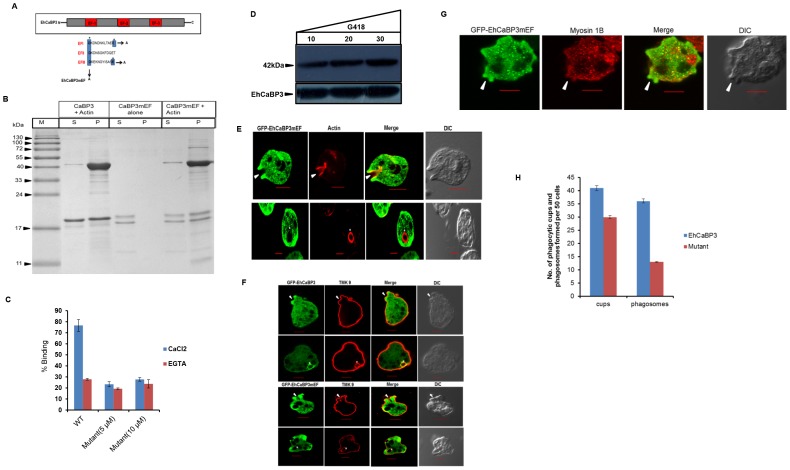
Generation and functional characterization of EhCaBP3mEF. (**A**) Schematic representation of EhCaBP3 indicating the three EF hand domains. The sequence of all the EF hands is shown and the first aspartate (D) of each EF hand and the last (E) amino acid residue was mutated to alanine (A) to generate CaBP3mEF mutant. (**B**) Cosedimentation assay of EhCaBP3 mutant with F-actin. Briefly 5 µM of F-actin was incubated with target proteins for 2 h. This was followed by ultracentrifugation and SDS-PAGE. (**C**) Solid-phase assay showing that CaBP3mEF was not able to bind G-actin efficiently as compared to wild type (WT). EhCaBP3mEF was added as indicated in the presence of 5 mM CaCl2 and 2 mM EGTA. EhCaBP3 (10 µM) was used as a control. (**D**) Cells expressing GFP-CaBP3mEF were maintained at different G418 concentrations (5, 10, 30 µg ml^−1^). Thirty microgram of the lysate was used for western blotting and protein was detected using anti-GFP antibody or anti-CaBP3 antibody. (**E**) Erythrophagocytosis of cells expressing GFP-CaBP3mEF. The slides were prepared as described in *Experimental procedures.* GFP-CaBP3mEF was stained with anti-GFP antibody (green) and TRITC-phalloidin (red) and viewed using confocal microscope. Upper panel: Confocal section of a representative cell showing enrichment of mutant in phagocytic cups and its co-localization with F-actin. Lower panel: Lack of EhCaBP3 mutant in phagosome. Bar represents 10 µm. Note the accumulation of GFP− CaBP3mEF in the phagocytic cup (arrow) and absent in phagosome (asterisk). (**F**) EhCaBP3mEF undergoing erythrophagocytosis followed by fixation. The cells were stained for EhCaBP3 or mutant (green) and TMK-9(red). Phagocytic cup represented by arrow heads and a phagosome by asterisk. Bar represents 10 µm. (**G**) Immunostaining of myosin 1B in the cells expressing GFP-EhCaBP3mEF. Cells were stained with anti-GFP antibody (green) and myosin 1B antibody (red). Arrow heads indicate the presence of EhCaBP3mEF and the absence of myosin 1B in the phagocytic cups. (**H**) Graph represents the number of phagocytic cups and phagosomes in over-expressed cell lines of EhCaBP3 and EhCaBP3mEF. Fifty cells were randomly selected for each experiment and counted the number of phagocytic cups and phagosomes present in all cells (blue, EhCaBP3 and red, EhCaBP3mEF).

We then investigated the importance of Ca^2+^ binding in the functioning of EhCaBP3. It was achieved by generating a mutant of EhCaBP3 which could not bind Ca^2+^ (EhCaBP3mEF). This was done by D→A and E→A mutagenesis respectively of the first D residue of all EF-hand motifs and last E residue of EF-I and EF-III (Figure 6A). The recombinant mutant protein did not bind Ca^2+^ as shown by ruthenium red staining (Figure S2). EhCaBP3mEF was checked for its ability to bind both F and G actin (Figure 6B and 6C). Polymerized actin co-sedimentation assay revealed that both wild type and mutant EhCaBP3 bound F-actin (Figure 6B). Binding to G-actin was carried out using a plate binding assay. EhCaBP3mEF did not bind G-actin unlike the wild type protein (Figure 6C) suggesting that binding of EhCaBP3 to G-actin requires involvement of Ca^2+^ whereas binding to F-actin does not.

### *In vivo* dynamics of EhCaBP3mEF

EhCaBP3mEF was also checked for its ability to get recruited in phagocytic cups and phagosomes. This was done by expressing a GFP-tagged mutant protein in *E. histolytica* cells (Figure 6D) and monitoring GFP as described in “materials and methods”. In order to mark the phagosomes properly, a plasma membrane marker (EhTMKB1–9) was used [34]. Immunofluorescence images revealed that while the mutant protein was observed in the cups (Figure 6E; upper panel), none of the phagosomes contained GFP-EhCaBP3mEF (Figures 6E and 6F; lower panel) unlike wild type protein (Figure 6F; upper panel). Further, actin was present in both cups and phagosomes in cells expressing the mutant protein (Figure 6E). However, myosin 1B enrichment and recruitment to those phagocytic cups was hampered where EhCaBP3mEF was present (Figure 6G), suggesting that Ca^2+^ is essential for recruitment of myosin 1B to phagocytic cups via EhCaBP3. This is supported by co-immunoprecipitation result as binding of EhCaBP3 to myosin 1B was inhibited in the presence of EGTA. Interestingly cells over expressing the mutant protein displayed only 20% reduction in phagocytic cups, while the reduction in phagosomes was 65% compared with cells over expressing the wild type EhCaBP3 (Figure 6H), suggesting a dominant negative effect of expression of the mutant protein. Since wild type EhCaBP3 continues to be expressed from the endogenous gene it is likely that these molecules help continuation of phagocytosis at a slower rate, even in the presence of EhCaBP3mEF.

### Downregulation of EhCaBP3 reduces the rate of phagocytosis

**Figure 7:**
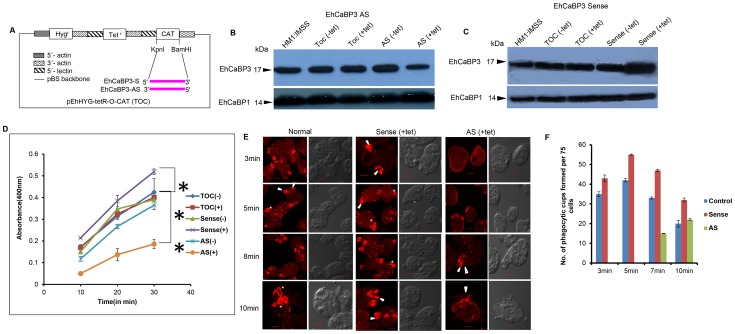
Downregulation of EhCaBP3 reduces the rate of phagocytosis. (**A**) Schematic representation of sense and anti-sense constructs. EhCaBP3 was cloned in the sense and the anti-sense orientation in BamH1 and Kpn1 sites of pEhHyg-TetR-O-CAT vector. (**B, C**) Western blot analysis of cell lines carrying the anti-Sense (**B**) and Sense (**C**) constructs. Thirty microgram of lysate from indicated cells in the presence and the absence of tetracycline were analyzed. Cells carrying without any construct were used as positive control and EhCaBP1 as loading control. (**D**) RBC uptake assay performed in cells expressing the sense and the anti-sense constructs in the presence and the absence of tetracycline. The experiment was repeated three times independently in triplicates. ANOVA test was used for statistical comparisons. P-values for * and ** are P<0.01 and P<0.001, respectively. #P>0.05. (**E**) Amoebic cells with and without indicated constructs were incubated with red blood cells (RBCs). These cells were then fixed and stained with TRITC-Phalloidin. The accumulation of actin at phagocytic cups were marked by a solid arrow heads and phagosomes were marked by asterisk. Open arrow heads mark attached RBCs at the site of phagocytosis. (**F**) Seventy five cells were randomly selected for each experiment and the number of phagocytic cups present in all cells were counted (blue, normal HM1: MSS; red, EhCaBP3S; green, EhCaBP3AS).

The results presented so far suggest that EhCaBP3 is associated with phagocytic machinery. In order to show whether it was also required for phagocytosis to occur, the level of EhCaBP3 was reduced by expressing specific antisense RNA. We have been able to down regulate expression of a number of genes using tetracycline-induced whole gene antisense RNA and this system was also employed to study the role of EhCaBP3 in phagocytosis [38]. The vector used and details of different constructs is shown in Figure 7A. On tetracycline addition the level of EhCaBP3 was significantly (55%) reduced in cells carrying antisense construct (EhCaBP3AS) as compared to the cells carrying only the vector (Figure 7B). This effect was specific as the amount of EhCaBP1 did not change. When EhCaBP3 gene was over expressed using the cloned gene in the sense orientation (EhCaBP3S) the amount of EhCaBP3 increased by 30% in the presence of 10 µg/ml of tetracycline (Figure 7C). *E. histolytica* cells carrying the sense and antisense constructs were then checked for erythrophagocytosis using a spectrophotometric assay. There was a 70% reduction in cells expressing EhCaBP3 antisense RNA (that is, in the presence of tetracycline) as compared with cells carrying only the vector in the presence of tetracycline, and cells carrying EhCaBP3 antisense construct in the absence of tetracycline. Over expression of EhCaBP3, that is addition of tetracycline to cells carrying a sense construct displayed an increase (40%) in erythrophagocytosis as compared to cells without tetracycline or vector containing cells in the presence of tetracycline (Figure 7D). The results of immunostaining of these cells are shown in Figure S3. The data showed that in cells expressing anti-sense RNA the cup formation was greatly reduced in the presence of tetracycline, while cup formation took place normally in cells expressing EhCaBP3 in the sense orientation with or without tetracycline.

Reduction in phagocytosis on down regulation of EhCaBP3 expression may be due to either a reduction in initiation, progression or scission of phagosome formation. It is also possible that all steps may be affected. In order to identify the site(s) affected, cells expressing EhCaBP3 antisense RNA were incubated with RBCs for indicated time and analysed by immunostaining. The results are shown in Figure 7E. In cells expressing EhCaBP3 in sense orientation many phagocytic cups were observed at about 3 min of incubation with RBC. However, the process of cup formation was delayed in antisense expressing cells. A few cups were visible only at about 8 min of incubation (Figure 7E). We also noticed that there was a defect in the closure of the cups to form phagosomes when EhCaBP3AS cells were incubated with RBC for 20 min (data not shown).The statistical analysis of the above data showed that cups appear in EhCaBP3AS cells at about 7 min after addition of RBC and there was a 58% reduction in the number of cups formed (Figure 7F). Interestingly cells over expressing EhCaBP3 consistently showed increased number of cups. It is also clear from Figure 7E that the amount of phalloidin staining in the cups is substantially less in EhCaBP3AS cells as compared to control cells suggesting that F-actin recruitment may also be affected. Quantitation of phalloidin staining in the cups showed 41% reduction in the intensity of F-actin in the phagocytic cups as compared to cells carrying only vector (Figure S4). This suggests that EhCaBP3 participates both in the initiation as well as closing stages during phagosome formation and that actin dynamics plays a critical role in EhCaBP3 function.

**Figure 8:**
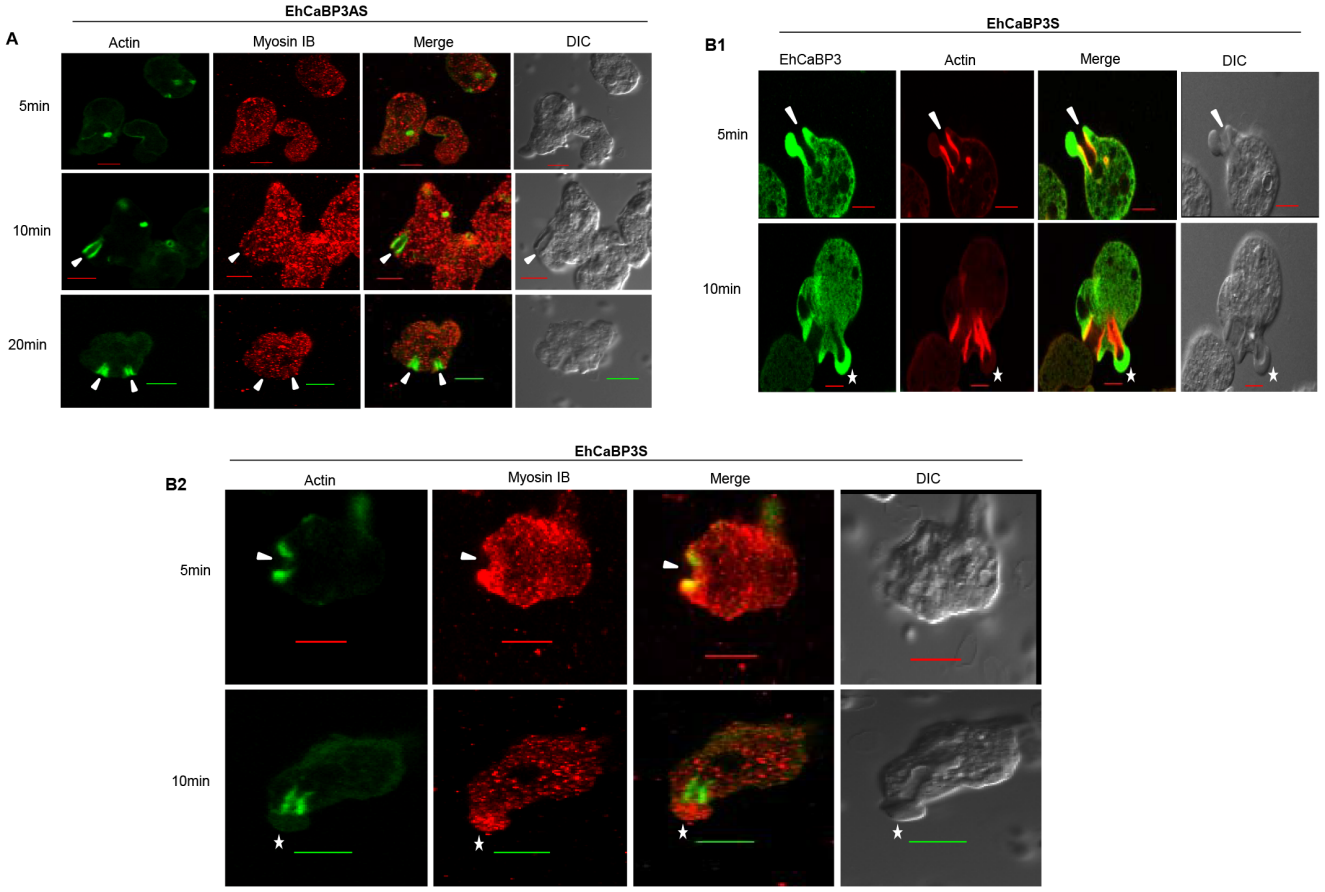
Distribution of myosin IB and actin in cells expressing EhCaBP3AS and EhCaBP3S. (**A**) Immunolocalization of myosin 1B and actin in EhCaBP3AS cell line. Cells were incubated with RBCs at indicated time points and then were fixed and stained with anti-myosin IB antibody (red) and FITC-phalloidin (green). (**B**) Immunolocalization of myosin 1B and actin in EhCaBP3S cell line. B1- Cells overexpressing EhCaBP3S were incubated with erythrocytes and were labeled with anti-EhCaBP3 antibody (Green) or TRITC-phalloidin (red). B2- Cells overexpressing EhCaBP3S were incubated with erythrocytes and were labeled with anti-myosin antibody (red) or FITC-phalloidin (green). Solid arrow heads indicate phagocytic cups and star shows the presence of CaBP3 and myosin 1B; and absence of actin at the site of cup closure.

We have observed colocalization of myosin IB with EhCaBP3 in phagocytic cups and phagosomes (Figure 4). Therefore the distribution of myosin IB in EhCaBP3AS was studied in order to further validate interaction of these two proteins during phagocytosis. The results showed the absence of myosin IB at the phagocytic cups even after 20 min of incubation with RBCs in cells expressing anti-sense RNA of EhCaBP3, suggesting that EhCaBP3 is required for recruitment of myosin IB (Figure 8A). We have also visualized distribution of EhCaBP3, myosin IB and actin in over expressing EhCaBP3S cells. EhCaBP3 and myosin IB were found to accumulate at the site of cup closure whereas actin was mainly present just at the neck (Figures 8B1 and B2; lower panel). There was no colocalization of EhCaBP3 and actin at the tip (Figure 8B1; lower panel), unlike myosin IB and EhCaBP3. Overall this data suggests that EhCaBP3 and myosin 1B are involved in phagocytosis and both these proteins may be needed for scission of vesicles.

**Figure 9:**
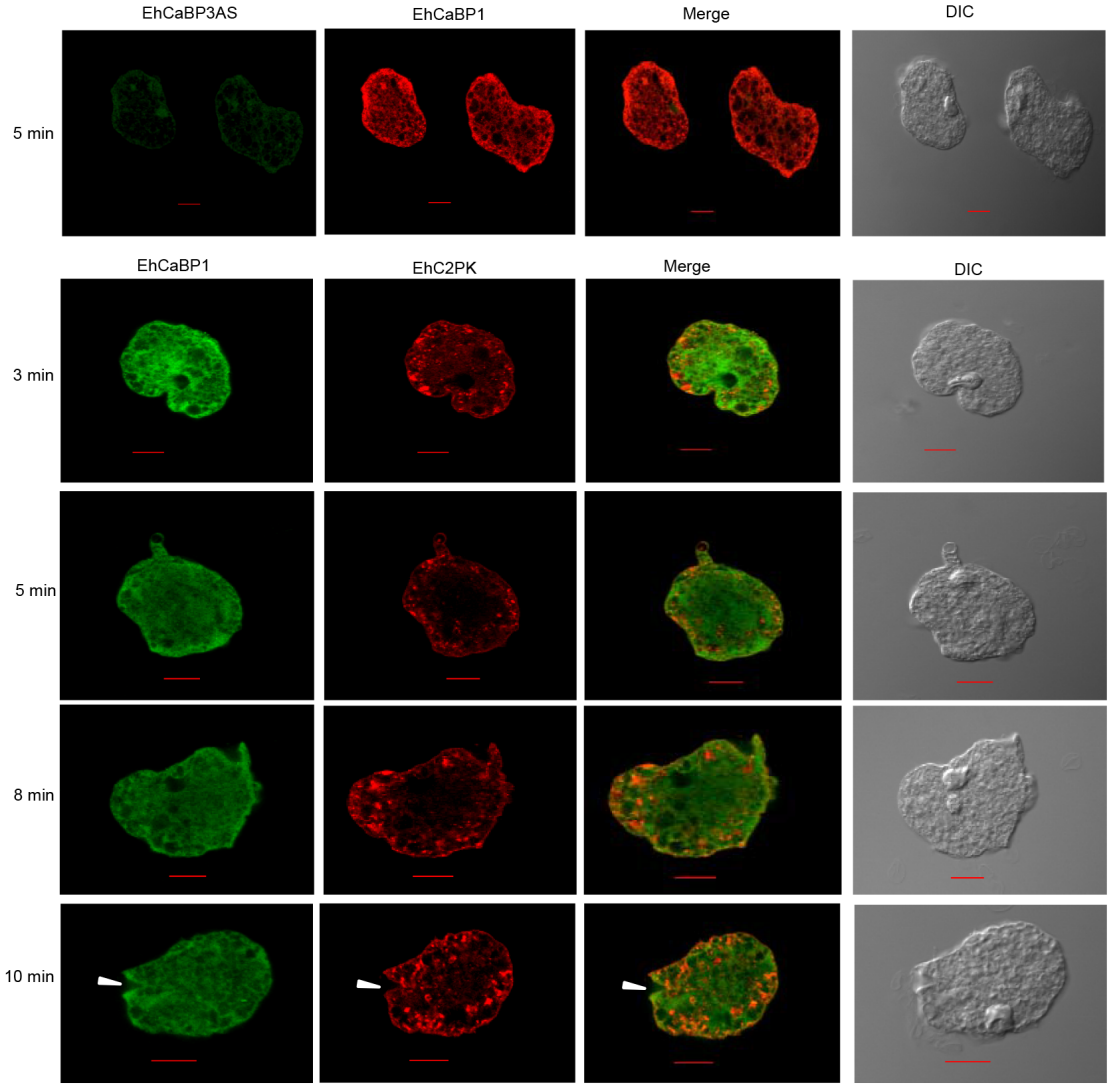
Localization of EhC2PK and EhCaBP1 in the anti-sense cell line. Cells were grown in the presence of tetracycline for 24 h and then incubated with RBCs at different time points. The cells were then fixed and immunostained with specific antibodies as described before. The antibodies used were anti-CaBP3 (green) and anti-CaBP1 (red) antibodies or anti-C2PK antibody (red) and anti-CaBP1 antibody (green) and viewed using Confocal Scanning Laser Microscope. Arrow head depicts the mild accumulation of EhCaBP1 and EhC2PK at the site of phagocytosis. Scale bar represents 10 µM.

To check whether EhCaBP3 level has any effect on recruitment of EhC2PK and EhCaBP1 in phagocytic cups; these proteins were immunostained in EhCaBP3 anti-sense cells (Figure 9). Reduced levels of EhCaBP1 and EhC2PK were observed in the phagocytic cups suggesting that EhCaBP3 may be involved in creating a macromolecular complex along with actin, EhC2PK and EhCaBP1.

### EhCaBP3 binds to lipids directly and recruits actin

We have shown earlier that EhC2PK binds liposomes in the presence of Ca^2+^ and recruits EhCaBP1 in a calcium dependent manner [34]. To test whether EhC2PK also recruits EhCaBP3 at the plasma membrane, we have used liposome sedimentation assay as described before [34]. The results are shown in Figure S5. The presence of a specific immunostained band in the pellet (which contains liposomes) is an indicator of interaction. Unlike EhCaBP1, EhCaBP3 bound liposomes directly without the involvement of EhC2PK in the presence of Ca^2+^. Actin was found in the pellet only when EhCaBP3 was present (Figure S5A). The interaction required the presence of Ca^+2^ as EGTA reduced the intensity of bands in western immunostaining. EhCaBP1 alone was not able to bind liposomes and EhC2PK-bound liposomes, as expected (Figure S5C, B). This suggests that EhCaBP3 alone can bind lipids, and consequently membranes, unlike EhCaBP1. These results are consistent with our previous finding that EhCaBP3 is also localized at the membrane in *E. histolytica* [35].

## Discussion

Regulated actin dynamics is required at different stages of phagocytosis and is achieved through participation of a number of molecules, many of which are actin binding proteins [12]. In mammalian system Arp2/3 complex, aphiphysin 2, coronin, cofilin, WASP and Scar (also called WAVE) are some of the molecules known to participate in regulating actin dynamics by manipulating different steps, such as nucleation, polymerization, bundling and depolymerization, including fragmentation of filaments [39]–[44]. Many processes involving actin dynamics, such as cell polarity, psuedopod formation and endocytosis in higher organisms have been studied in detail and the molecular mechanisms mediating different steps of actin dynamics have been worked out [45], [46]. However, the mechanism of initiation of phagocytosis is understood only in a few systems, of which the best studied, is opsonisation involving Fc receptors [47]. Our laboratory has shown that a C2 domain-containing protein kinase EhC2PK along with a calcium binding protein EhCaBP1 is involved in initiating a signal transduction pathway that eventually results in phagocytosis of RBCs in the protist parasite *E. histolytica* [34]. EhCaBP1 helps in recruiting actin at the site of phagocytosis by bridging with EhC2PK, a Ca^2+^-dependent membrane binding protein. This is one of the first examples of direct involvement of a calcium binding protein in actin dynamics and initiation of an endocytic process. In this report we show that *E. histolytica* erythrophagocytosis requires participation of yet another calcium binding protein EhCaBP3. Our results suggest that unlike EhCaBP1 which acts only at the initiation stage of phagocytosis, EhCaBP3 is likely to participate in both initiation and phagosome closure stages. It also appears from our results that the proposed mechanism may not be applicable for RBC phagocytosis alone, but also applicable in phagocytosis of CHO cells, though the detailed mechanisms may be somewhat different.

A number of our observations support the conclusion that EhCaBP3 is involved in phagocytosis. Firstly, EhCaBP3 was observed in phagocytic cups and phagosomes by fluorescence imaging of both fixed and live cells. Secondly, the rate and extent of phagocytosis was greatly reduced in cells where EhCaBP3 expression was down regulated by antisense RNA, and finally, over expression of a Ca^2+^ binding- defective mutant of EhCaBP3 reduced the rate of phagosome formation showing a dominant negative phenotype. Though the involvement of EhCaBP3 in the initiation of phagocytosis appears to be similar to that of EhCaBP1, there are important differences in their chemical and biological properties. For example, Ca^2+^ binding affinities of the two molecules are different. The overall Ca^2+^ binding affinity of EhCaBP1 was more than 700 fold that of EhCaBP3, and their dissociation constants (K_d_) were 1.3 nM and 1.85 µM respectively [19], [35]. This would indicate that the two proteins function optimally at different Ca^2+^ concentrations. However, we are not in a position to correlate actual local transient Ca^2+^ concentrations during phagocytosis with the function and Ca^2+^ binding properties of the two proteins due to lack of data about Ca^2+^ concentrations in *E. histolytica*. However, we can speculate that attachment of RBC to the surface of *E. histolytica* generates a local Ca^2+^ spike. EhCaBP3 and EhCaBP1 are likely to get activated at different stages of a spike when Ca^2+^ concentration can vary about 2 orders of magnitude resulting in sequential activation of the two EhCaBPs. However, we do not have at present any evidence in support of this. Further the two proteins are functionally different since EhCaBP1 is absent in newly formed phagosomes, while EhCaBP3 is present. This is an indication that EhCaBP3 may be participating in the process of phagosome closure.

The presence of EhCaBP3 along with myosin IB at the tip of membranes before closure to form phagosomes strongly suggests that EhCaBP3 along with myosin IB may be involved in psuedopod extension, phagosome closure and finally release of the vesicle into the cytoplasm. Association of myosin 1B and EhCaBP3 has also been validated by a pull down assay. This appears to be similar to a mammalian long tail class 1 myosin that also localizes to phagosomes at late stages and participates in phagosome closure [48]. Further, transient localization of class 1 myosin to phagocytic cups has also been observed in *Acanthamoeba,* and in yeast myosin 1 facilitates different events of endocytosis, such as membrane fusion and vesicle scission [49], [50]. Myosins are also known to manipulate dynamics of actin filaments [51]. However, the interplay between myosins and actin in filament dynamics in relation to phagocytosis, psuedopod formation and motility in *E. histolytica* is not yet understood. Both EhCaBP1 and EhCaBP3 can bind G- and F-actin directly. However, the effect of this binding translates into different biochemical changes. EhCaBP1 alters bundling properties of actin filaments without changing polymerization [21]. On the other hand, as shown here, EhCaBP3 enhances polymerization in addition to enhancing bundling formation. Together these two calcium binding proteins modulate dynamic properties of actin cytoskeleton, a unique feature not seen in any other system. Though the effects of EhCaBP1 and EhCaBP3 on actin polymerization and bundling were studied *in vitro*, we believe that the same properties are likely to be seen *in vivo*. Our assumptions are based on colocalization of actin filaments with these two proteins during phagocytosis and the observation that there is a reduction in phagocytosis when either the expression is reduced or mutant proteins are present. The reduction in phagocytosis is likely to be due to a defect in actin filament formations and this has been seen in reduced amount of F-actin in the phagocytic cups of EhCaBP3AS cells. It is also likely that the process of initiation is achieved through multiple steps and that EhCaBP3 interacts with as yet unknown molecules (other than actin) that participate in these steps. The molecular details of sequential changes in the state of actin, and the possible recruitment of other proteins by the CaBPs need to be worked out. It is not clear how EhCaBP1 moves out of the phagocytic cups before phagosome closure while EhCaBP3 does not. We suspect that other proteins, such as myosin IB may be involved as both myosin IB and EhCaBP3 were seen at the tips before closure.

Since EhCaBP3 is a small Ca^2+^ binding protein and only contains Ca^2+^- binding EF-hand motifs, it is expected that its function must be executed through binding of Ca^2+^. However, we observed that EhCaBP3mEF, a mutant of EhCaBP3 that could not bind Ca^2+^, is present in the phagocytic cups and over expression of this mutant protein led to only a small reduction in phagocytic cup formation. This is not surprising as the mutant protein is capable of binding F-actin and can cause bundling of actin similar to the wild type protein. The reasons for reduction in cup formation, though small, as compared to phagosome formation, on overexpression of the mutant protein in spite of being recruited in the cups, may be due to involvement of other proteins. We need to characterize the initiation complex and identify all the players before we can answer this question. However, it is clear that Ca^2+^ binding of EhCaBP3 is necessary for phagosome formation as only Ca^2+^-bound form of EhCaBP3 interacts with myosin 1B, and the latter's recruitment in phagocytic cups requires the wild type protein. Therefore, it appears that Ca^2+^ has multiple facilitators in the form of different CaBPs, and a large number of different species (Ca^2+^ bound and free forms) participate at different steps in the process of phagocytosis. We are beginning to understand some of the steps as outlined here.

The mechanism of recruitment of EhCaBP3 during the process of initiation of phagocytosis is not clear. Since it does not bind EhC2PK it may require participation of yet other unknown molecule(s). Alternately, molecules that are present in the membrane or may be recruited to the membrane due to changes in local Ca^2+^ concentration could form initiation complexes along with EhC2PK, EhCaBP1 and actin, along with other participants. Support for this comes from our observations that EhCaBP3 can bind liposomes in the presence of Ca^2+^ and can also form a complex with liposomes and actin. Interestingly the requirement of a complex formation involving EhCaBP1, EhC2PK and EhCaBP3 for initiation of phagocytosis is evident from the reduced recruitment of both EhCaBP1 and EhC2PK in EhCaBP3 down regulated cells. However, it is not clear if EhCaBP3 present in phagocytic cups migrates from other parts of the membrane or from a pool of membrane-bound EhCaBP3, or from the cytoplasmic pool. Further studies are needed to work out the detailed mechanisms including the pathway involved in formation of a multimeric complex of these proteins.

EhCaBP3 is likely to participate in multiple processes other than phagocytosis and actin mobilization. It is also present in the nucleus and the function of nuclear EhCaBP3 is not clear. Our studies show that the process of RBC phagocytosis in the human parasite *E. histolytica* follows a unique mechanism involving a number of molecules that have been identified only in this organism. Deciphering this pathway will be highly useful in understanding evolution of phagocytic mechanisms in eukaryotic cells, as *E. histolytica* is an early branching eukaryote. Moreover, phagocytosis is essential for the growth and survival of this parasite and blocking this process leads to inhibition of cellular proliferation. Therefore, unique molecules involved in the pathway could be potential targets for developing newer drugs.

## Materials and Methods

### Growth conditions, transfection and selection

*E. histolytica* stain HM1: IMSS and the transformants were maintained and grown in TYI-S-33 medium as described before [52]. Neomycin or Hygromycin (Sigma) were added at 10 µg ml^−1^ for maintaining transgenic cell lines as indicated.

Transfection was performed by electroporation. Mid-log phase cells were harvested and washed first by PBS and then cytomix buffer (10 mM K_2_HPO_4_/KH_2_PO_4_ (pH 7.6), 120 mM KCl, 0.15 mM CaCl_2_, 25 mM HEPES (pH 7.4), 2 mM EGTA, 5 mM MgCl_2_). The washed cells were then re-suspended in 0.8 ml of cytomix buffer containing 4 mM adenosine triphosphate, 10 mM glutathione and 200 µg of plasmid DNA. The suspension was then subjected to two consecutive pulses of 3,000 V cm^−1^(1.2 kV) at 25 µF (Bio-Rad, electroporator). The transfectants were initially allowed to grow without any selection for 48 h. Selection was carried out by adding G418 or hygromycin B (10 µg ml^−1^) depending on the plasmid used.

### Construction of different vectors

EhCaBP3 gene was cloned in the BamH1 site of pEh-Neo-GFP vector. The vector has been previously constructed (Gullien, unpublished) by cloning the GFP mut3 allele of GFP [53] in the unique BamH1 site of the pExEhNeo plasmid [54]. Calcium binding defective mutant was also cloned in the pEh-Neo-GFP vector at the C-terminus of GFP. The CAT gene of the shuttle vector pEhHYG-tetR-O-CAT [55] was excised using KpnI and BamHI and EhCaBP3 gene was inserted in its place in either the sense or the antisense orientation. The sequences of oligonucleotides used for making the above stated constructs are described in the Supplementary Table S1. Standard molecular techniques were used for making all these constructs.

### Co-sedimentation assay

Co-sedimentation assay was carried out following published conditions [21]. Briefly, 5 µM of rabbit muscle G-actin (Sigma) was polymerized in a polymerization buffer containing 100 mM KCl and 2 mM MgCl_2_ at room temperature for 1 h. After polymerization, actin was mixed with 1 mM ATP and appropriate target protein (5 µ M) in a total volume of 150 µl of G-buffer (10 mM Tris-Cl, pH 7.5, 2 mM CaCl_2_, 2.5 mM β-Mercaptoethanol, 0.5 M KCl, 10 mM MgCl_2_) and incubated for 2 h at room temperature. The samples were centrifuged at 100,000 *g* for 45 min at 4°C. The supernatant (one-fourth of the total) and pellet fractions (total) were analysed by 14% SDS-PAGE followed by Coomassie blue staining. In addition to WT (EhCaBP3), mutant (EhCaBP3mEF), EhCaBP1 and EhCaBP2 were also used as positive and negative controls respectively.

### Solid-phase assay

Solid phase G-actin binding assay was carried out as described before [21]. Briefly, different wells of a 96-well plate were coated with 5 µM G-actin in PBS overnight at 4°C and were blocked with 3% BSA in PBS for an additional 24 h. After washing with PBS-T (PBS containing (0.05% Tween-20), EhCaBP3 and CaBP3mEF were added to the wells in duplicates at concentrations ranging from 0.5 µM to 10 µM. Bound protein was detected with anti-EhCaBP3 antibody followed by HRPO-linked anti-rabbit IgG using the colorimetric substrate TMB (Sigma). The absorbance was monitored at 405 nm with a microplate reader (Bio-Rad, USA) after stopping the reaction with 2 N H_2_SO_4_. The reaction was carried out in the presence of 5 mM CaCl_2_ or 2 mM EGTA as indicated.

### Actin polymerization assay

Polymerization assay was done as per the protocol supplied by the manufacturer (www.cytoskeleton.com). Polymerization of actin was monitored by an increase in fluorescence of pyrene-labeled actin (cytoskeleton, USA) with excitation at 366 nm and emission at 407 nm. The assays were carried out at 20°C in a Safas Flx spectrofluorimeter. A 100 µl sample containing 3 µM pyrene-labelled G-actin, was saturated with increasing concentration of EhCaBP3 (3 µM, 5 µM, 10 µM and 15 µM). EhCaBP1 (5 µM) was used as a control and the reactions were carried out in polymerization buffer (5 mM Tris-HCl, pH 7.5, 1 mM dithiothreitol (DTT), 0.2 mM ATP, 0.1 mM CaCl_2_, 0.01% NaN3,0.1 M KCl and 1 mM MgCl_2_).

### Actin bundling assay and erythrophagocytosis

The assays were carried as described before [21] and details are given in Text S1.

### Immunoprecipitation

CNBr-activated Sepharose-4B (1 g, Pharmacia) was conjugated with anti-EhCaBP3 antibody following a protocol supplied by the manufacturer. Briefly, crude immunoglobulins were collected from the immunized serum using 40% ammonium sulphate and subsequently dialysed in coupling buffer (bicarbonate buffer). Usually, 10 mg protein was added per gram of resin. The resin was mixed gently for 18 h at 4°C. After coupling the coupled resin was processed as per the manual provided by the manufacturer. The conjugated Sepharose beads were incubated with *E. histolytica* lysate for 6 h at 4°C. The beads were then washed with wash buffer (10 mM Tris-Cl (pH 7.5), 150 mM NaCl, 1 mM imidazole, 1 mM magnesium acetate, 2 mM β-ME and protease inhibitor cocktail) three times. Ca^2+^ and EGTA were maintained throughout the process as required. After incubation the beads were washed sequentially with 60 mM Tris-Cl (pH 6.8), 100 mM NaCl and with 60 mM Tris-Cl (pH 6.8). The pellet was suspended in 2× SDS polyacrylamide gel electrophoresis (PAGE) buffer and boiled for 5 min followed by centrifugation for 5 min. The proteins were then analyzed by western blotting.

### Immunofluorescence labelling

*E. histolytica* cells were labelled for immunofluorescent imaging following methods described before [21]. Cells were first washed with PBS and incomplete TYI-S-33 medium, and then resuspended in the same medium before transferring onto acetone-cleaned coverslips placed in a Petri dish. The cells were allowed to adhere for 10 min at 37°C and then were fixed with 3.7% paraformaldehyde (PFA) for 30 min at 37°C after removing the culture medium. The fixed cells were then permeabilized with 0.1%Triton X-100/PBS for 5 min. Additional treatment using chilled methanol (−20°C) for 3 min was needed for staining myosin IB. Fixed cells were then washed with PBS and quenched with 50 mM NH_4_Cl for 30 min at 37°C, followed by blocking with 1%BSA-PBS for 1 h. The cells were then incubated with primary antibody for 1 h at 37°C, followed by washing with PBS and 1%BSA-PBS before incubation with secondary antibody for 45 min at 37°C. When F-actin was labelled with phalloidin, the methanol step was omitted. Antibody dilutions used were: EhCaBP3 at 1∶50 (purified antibody), EhCaBP1 at 1∶200, phalloidin (Sigma; 1 mg/ml) at 1∶250, GFP monoclonal (Molecular Probes, Cat no. A11120) at 1∶250, myosin IB at 1∶30 [56], anti-rabbit or mice Alexa 488 (Molecular Probes, Catalogue No. A-11008 or A-11001) at 1∶200, anti-rabbit or mice Alexa 555 (Molecular Probes, Cat. No. A-21428 or A-21422) at 1∶300. The preparations were further washed with PBS and mounted on a glass slide using DABCO [1, 4-diazbicyclo (2, 2, 2) octane (Sigma) 10 mg/ml in 80% glycerol]. The edges of the coverslips were sealed with nail-paint to avoid drying. Confocal images were visualized by using an Olympus Fluoview FV1000 laser scanning microscope.

CHO cells were stained for 30 min with 20 mM Cell tracker orange dye (Molecular probes, Eugene, OR) in F12 medium containing 10% FCS. After staining, CHO cells were washed three times with fresh BI-S-33 medium, and approximately 4×10^5^ CHO cells were incubated with 2×10^5^ cells of amoeba expressing GFP-CaBP3 for indicated time points at 37°C in 500 µl of TYI-33 medium.

### Time-lapse imaging

The cells expressing GFP-EhCaBP3 were plated onto a 35 mm Mat Tek glass bottom culture dish (MatTek Corporation) at 37°C. The medium was then removed after the cells got settled at the bottom and the glass chamber was filled with pre-warmed PBS. The dish was kept on a platform with a temperature controller to maintain temperature at 37°C. High-resolution fluorescent time-lapse imaging (Nikon A1R) of a moving and phagocytosing amoeba was performed. The images were captured at 8 s interval and 60× objective was used. The raw images were processed using NIS element 3.20 or Image J software available freely on the web (http://rsb.info.nih.gov/ij/).

### Western blotting

Samples were separated on a 14% SDS–PAGE and the gel was then transferred on to a polyvinylidine fluoride (PVDF) membrane by semi-dry method and processed using standard protocols. The antigens were detected with polyclonal anti-GFP (1∶5000, Molecular probes; Cat. No. A6455) or anti-EhCaBP1 or EhCaBP3 raised in mice and rabbits (1∶5000, raised in our laboratory), followed by secondary anti-rabbit and anti-mice immunoglobulins conjugated to HRPO at 1∶10,000 dilution (Sigma, Cat No's A6667 or A2554). ECL reagents were used for visualization (Millipore). The concentration of proteins in a sample was estimated by bicinchoninic acid (BCA) assay using BSA as a standard.

### Ruthenium red staining

The assay was carried as described before [57] and details are given in Text S1.

### Liposome-binding assay

The liposomes were prepared as described by Avanti Polar Lipid, Inc. http://avantilipids.com. The proteins were incubated with liposomes in binding buffer (Tris-Cl (pH 7.5) 10 mM, β-ME 0.25 mM, NaCl 50 mM). CaCl_2_ and EGTA were used at 2 mM and 5 mM respectively at 37°C for 2 h with intermittent tapping. The liposomes were centrifuged at 18,000 *g* for 30 min, followed by washing with binding buffer to remove the nonspecific-binding proteins. Liposomes were than dissolved in SDS buffer and separated on SDS–PAGE. Specific proteins were detected by western blotting. For actin-binding assay the liposomes were incubated in polymerization buffer (Tris-Cl (pH 7.5) 10 mM, MgCl_2_ 2 mM, KCl 50 mM, ATP 2.5 mM, β-ME 2.5 mM) with EhCaBP3, and actin.

#### Statistical analysis

Statistical comparisons were made using a one-way ANOVA test. Experimental values were reported as the means ± s.e. Differences in mean values were considered significant at *P*<0.05. All calculations of statistical significance were made using the GraphPad InStat software package (GraphPad).

### Ethics statement

Both mice and rabbits used for generation of antibodies were approved by the Institutional Animal Ethics Committee (IAEC), Jawaharlal Nehru University (IAEC Code No.: 18/2010).

All animal experimentations were performed according to the National Regulatory Guidelines issued by CPSEA (Committee for the Purpose of Supervision of Experiments on Animals), Ministry of Environment and Forest, Govt. of India.

## Supporting Information

Figure S1 Localization of GFP–EhCaBP3 during CHO cell phagocytosis. CHO cells were labelled with Cell tracker orange and added to the cells expressing GFP–EhCaBP3 (approximately 1∶2 ratio). Cells were fixed and labelled with anti-GFP antibody, followed by Alexa-mouse 488. Arrows depict the phagocytic cups and asterisk indicates phagosomes containing CHO cells. Scale bar represents 20 µm.
(PPT)

Figure S2 Calcium ability test by Ruthenium red staining. Purified recombinant proteins were blotted onto PVDF membrane, followed by washing. Then 25 µg ml^−1^ ruthenium red stain was added. EhCaBP1 and BSA were used as positive and negative controls respectively.
(PPT)

Figure S3 Cells expressing the anti-sense (A–B) and the sense (C–D) constructs in presence and absence of tetracycline were incubated with RBCs for 7 min. The cells were then fixed and stained with Alexa 488 (EhCaBP3) and TRITC-phalloidin (actin). Solid arrow heads represent the phagocytic cups, open arrow shows attached RBC. *(Scale bar, 10 µm; DIC, differential interference contrast).*
(PPT)

Figure S4 Quantitation of phalloidin staining in anti-sense phagocytosing cells. Intensity of F-actin was measured at multiple locations in the cytosol and in phagocytic cups. Average relative intensity of phagocytic cups was computed by taking the signal from cytosol as 100%. (N = 10, bars represent standard error).
(PPT)

Figure S5 Binding of EhCaBP3 to phosphatidylserine. Presence of specific proteins in the pellet (P), that is, liposome fraction indicates binding. Proteins that do not bind remain in the soluble fraction (S). (**A**) Purified EhCaBP3 and actin were incubated with phosphatidylserine (PS) liposomes in the presence and the absence of Ca^2+^. (**B**) Purified GST-EhC2PK and EhCaBP1 were incubated with liposomes in presence and absence of Ca^2+^. (**C–D**) EhCaBP1 and actin were incubated with liposomes.
(PPT)

Movie S1 Live cell imaging of amoebic cells expressing GFP-EhCaBP3. The movie represents the time sequence of a moving amoeba expressing GFP-EhCaBP3. Note the enrichment of EhCaBP3 within 8 s at the site of RBC attachment to the cell surface of amoeba. Bar represents 5 µm.
(AVI)

Table S1 List of oligonucleotides used in the study.
(DOC)

Text S1 Supplementary methods.
(DOC)

## References

[1] WHO/PAHO/UNESCO report. A consultation with experts on amoebiasis. Mexico City, Mexico 28–29 January, 1997. Epidemiol Bull 18: 13–14. 9197085

[2] Stanley SL Jr (2003) Amoebiasis. Lancet 361: 1025–1034. 12660071 10.1016/S0140-6736(03)12830-9

[3] Orozco E, Guarneros G, Martinez-Palomo A, Sanchez T (1983) *Entamoeba histolytica*. Phagocytosis as a virulence factor. J Exp Med 158: 1511–1521. 6313842 10.1084/jem.158.5.1511 PMC2187140

[4] Tsutsumi V, Ramirez-Rosales A, Lanz-Mendoza H, Shibayama M, Chavez B, et al (1992) Entamoeba histolytica: erythrophagocytosis, collagenolysis, and liver abscess production as virulence markers. Trans R Soc Trop Med Hyg 86: 170–172. 1440779 10.1016/0035-9203(92)90555-q

[5] Aderem A, Underhill DM (1999) Mechanisms of phagocytosis in macrophages. Annu Rev Immunol 17: 593–623. 10358769 10.1146/annurev.immunol.17.1.593

[6] Kwiatkowska K, Sobota A (1999) Signaling pathways in phagocytosis. Bioessays 21: 422–431. 10376013 10.1002/(SICI)1521-1878(199905)21:5<422::AID-BIES9>3.0.CO;2-#

[7] Tjelle TE, Lovdal T, Berg T (2000) Phagosome dynamics and function. Bioessays 22: 255–263. 10684585 10.1002/(SICI)1521-1878(200003)22:3<255::AID-BIES7>3.0.CO;2-R

[8] Bengtsson T, Jaconi ME, Gustafson M, Magnusson KE, Theler JM, et al (1993) Actin dynamics in human neutrophils during adhesion and phagocytosis is controlled by changes in intracellular free calcium. Eur J Cell Biol 62: 49–58. 8269978

[9] Holm A, Tejle K, Magnusson KE, Descoteaux A, Rasmusson B (2001) *Leishmania donovani* lipophosphoglycan causes periphagosomal actin accumulation: correlation with impaired translocation of PKCalpha and defective phagosome maturation. Cell Microbiol 3: 439–447. 11437830 10.1046/j.1462-5822.2001.00127.x

[10] Castellano F, Montcourrier P, Guillemot JC, Gouin E, Machesky L, et al (1999) Inducible recruitment of Cdc42 or WASP to a cell-surface receptor triggers actin polymerization and filopodium formation. Curr Biol 9: 351–360. 10209117 10.1016/s0960-9822(99)80161-4

[11] Jaconi ME, Lew DP, Carpentier JL, Magnusson KE, Sjogren M, et al (1990) Cytosolic free calcium elevation mediates the phagosome-lysosome fusion during phagocytosis in human neutrophils. J Cell Biol 110: 1555–1564. 2110568 10.1083/jcb.110.5.1555 PMC2200167

[12] Castellano F, Chavrier P, Caron E (2001) Actin dynamics during phagocytosis. Semin Immunol 13: 347–355. 11708890 10.1006/smim.2001.0331

[13] Goley ED, Welch MD (2006) The ARP2/3 complex: an actin nucleator comes of age. Nat Rev Mol Cell Biol 7: 713–726. 16990851 10.1038/nrm2026

[14] Pantaloni D, Carlier MF (1993) How profilin promotes actin filament assembly in the presence of thymosin beta 4. Cell 75: 1007–1014. 8252614 10.1016/0092-8674(93)90544-z

[15] Lappalainen P, Kessels MM, Cope MJ, Drubin DG (1998) The ADF homology (ADF-H) domain: a highly exploited actin-binding module. Mol Biol Cell 9: 1951–1959. 9693358 10.1091/mbc.9.8.1951 PMC25446

[16] Lew DP, Andersson T, Hed J, Di Virgilio F, Pozzan T, et al (1985) Ca2+-dependent and Ca2+-independent phagocytosis in human neutrophils. Nature 315: 509–511. 3158824 10.1038/315509a0

[17] Stendahl O, Krause KH, Krischer J, Jerstrom P, Theler JM, et al (1994) Redistribution of intracellular Ca2+ stores during phagocytosis in human neutrophils. Science 265: 1439–1441. 8073285 10.1126/science.8073285

[18] Vieira OV, Botelho RJ, Grinstein S (2002) Phagosome maturation: aging gracefully. Biochem J 366: 689–704. 12061891 10.1042/BJ20020691 PMC1222826

[19] Jain R, Santi-Rocca J, Padhan N, Bhattacharya S, Guillen N, et al (2008) Calcium-binding protein 1 of *Entamoeba histolytica* transiently associates with phagocytic cups in a calcium-independent manner. Cell Microbiol 10: 1373–1389. 18341598 10.1111/j.1462-5822.2008.01134.x

[20] Ohsawa K, Imai Y, Kanazawa H, Sasaki Y, Kohsaka S (2000) Involvement of Iba1 in membrane ruffling and phagocytosis of macrophages/microglia. J Cell Sci 113 (Pt 17) 3073–3084. 10934045 10.1242/jcs.113.17.3073

[21] Sahoo N, Labruyere E, Bhattacharya S, Sen P, Guillen N, et al (2004) Calcium binding protein 1 of the protozoan parasite Entamoeba histolytica interacts with actin and is involved in cytoskeleton dynamics. J Cell Sci 117: 3625–3634. 15252130 10.1242/jcs.01198

[22] Hayes MJ, Shao D, Bailly M, Moss SE (2006) Regulation of actin dynamics by annexin 2. Embo J 25: 1816–1826. 16601677 10.1038/sj.emboj.7601078 PMC1456940

[23] Fechheimer M, Taylor DL (1984) Isolation and characterization of a 30,000-dalton calcium-sensitive actin cross-linking protein from Dictyostelium discoideum. J Biol Chem 259: 4514–4520. 6707016

[24] Brown SS, Yamamoto K, Spudich JA (1982) A 40,000-dalton protein from *Dictyostelium discoideum* affects assembly properties of actin in a Ca2+-dependent manner. J Cell Biol 93: 205–210. 7068756 10.1083/jcb.93.1.205 PMC2112095

[25] Yamamoto K, Pardee JD, Reidler J, Stryer L, Spudich JA (1982) Mechanism of interaction of Dictyostelium severin with actin filaments. J Cell Biol 95: 711–719. 6897549 10.1083/jcb.95.3.711 PMC2112927

[26] Schroeter M, Chalovich JM (2004) Ca^2+^-calmodulin regulates fesselin-induced actin polymerization. Biochemistry 43: 13875–13882. 15504050 10.1021/bi0487490

[27] Dharamsi A, Tessarolo D, Coukell B, Pun J (2000) CBP1 associates with the *Dictyostelium* cytoskeleton and is important for normal cell aggregation under certain developmental conditions. Exp Cell Res 258: 298–309. 10896781 10.1006/excr.2000.4950

[28] Fujimoto LM, Roth R, Heuser JE, Schmid SL (2000) Actin assembly plays a variable, but not obligatory role in receptor-mediated endocytosis in mammalian cells. Traffic 1: 161–171. 11208096 10.1034/j.1600-0854.2000.010208.x

[29] van Zon JS, Tzircotis G, Caron E, Howard M (2009) A mechanical bottleneck explains the variation in cup growth during FcgammaR phagocytosis. Mol Syst Biol 5: 298. 19690567 10.1038/msb.2009.59 PMC2736656

[30] Mitchell MA, Huang MM, Chien P, Indik ZK, Pan XQ, et al (1994) Substitutions and deletions in the cytoplasmic domain of the phagocytic receptor Fc gamma RIIA: effect on receptor tyrosine phosphorylation and phagocytosis. Blood 84: 1753–1759. 7521687

[31] Odin JA, Edberg JC, Painter CJ, Kimberly RP, Unkeless JC (1991) Regulation of phagocytosis and [Ca2+]i flux by distinct regions of an Fc receptor. Science 254: 1785–1788. 1837175 10.1126/science.1837175

[32] Tollis S, Dart AE, Tzircotis G, Endres RG (2010) The zipper mechanism in phagocytosis: energetic requirements and variability in phagocytic cup shape. BMC Syst Biol 4: 149. 21059234 10.1186/1752-0509-4-149 PMC2991294

[33] Bhattacharya A, Padhan N, Jain R, Bhattacharya S (2006) Calcium-binding proteins of Entamoeba histolytica. Arch Med Res 37: 221–225. 16380322 10.1016/j.arcmed.2005.10.002

[34] Somlata, Bhattacharya S, Bhattacharya A (2011) A C2 domain protein kinase initiates phagocytosis in the protozoan parasite Entamoeba histolytica. Nat Commun 2: 230. 21407196 10.1038/ncomms1199

[35] Rout AK, Padhan N, Barnwal RP, Bhattacharya A, Chary KV (2011) Calmodulin-like Protein from *Entamoeba histolytica*: Solution Structure and Calcium-Binding Properties of a Partially Folded Protein. Biochemistry 50: 181–93. 21114322 10.1021/bi101411q

[36] Nakada-Tsukui K, Okada H, Mitra BN, Nozaki T (2009) Phosphatidylinositol-phosphates mediate cytoskeletal reorganization during phagocytosis via a unique modular protein consisting of RhoGEF/DH and FYVE domains in the parasitic protozoon *Entamoeba histolytica* . Cell Microbiol 11: 1471–1491. 19496789 10.1111/j.1462-5822.2009.01341.x

[37] Voigt H, Guillen N (1999) New insights into the role of the cytoskeleton in phagocytosis of *Entamoeba histolytica* . Cell Microbiol 1: 195–203. 11207552 10.1046/j.1462-5822.1999.00021.x

[38] Sahoo N, Bhattacharya S, Bhattacharya A (2003) Blocking the expression of a calcium binding protein of the protozoan parasite *Entamoeba histolytica* by tetracycline regulatable antisense-RNA. Mol Biochem Parasitol 126: 281–284. 12615327 10.1016/s0166-6851(02)00284-0

[39] May RC, Caron E, Hall A, Machesky LM (2000) Involvement of the Arp2/3 complex in phagocytosis mediated by FcgammaR or CR3. Nat Cell Biol 2: 246–248. 10783245 10.1038/35008673

[40] Lorenzi R, Brickell PM, Katz DR, Kinnon C, Thrasher AJ (2000) Wiskott-Aldrich syndrome protein is necessary for efficient IgG-mediated phagocytosis. Blood 95: 2943–2946. 10779443

[41] Yan M, Collins RF, Grinstein S, Trimble WS (2005) Coronin-1 function is required for phagosome formation. Mol Biol Cell 16: 3077–3087. 15829569 10.1091/mbc.E04-11-0989 PMC1165393

[42] Yamada H, Ohashi E, Abe T, Kusumi N, Li SA, et al (2007) Amphiphysin 1 is important for actin polymerization during phagocytosis. Mol Biol Cell 18: 4669–4680. 17855509 10.1091/mbc.E07-04-0296 PMC2043535

[43] Abou-Kheir W, Isaac B, Yamaguchi H, Cox D (2008) Membrane targeting of WAVE2 is not sufficient for WAVE2-dependent actin polymerization: a role for IRSp53 in mediating the interaction between Rac and WAVE2. J Cell Sci 121: 379–390. 18198193 10.1242/jcs.010272 PMC2749557

[44] Poukkula M, Kremneva E, Serlachius M, Lappalainen P (2011) Actin-depolymerizing factor homology domain: a conserved fold performing diverse roles in cytoskeletal dynamics. Cytoskeleton (Hoboken) 68: 471–490. 21850706 10.1002/cm.20530

[45] Harrison RE, Grinstein S (2002) Phagocytosis and the microtubule cytoskeleton. Biochem Cell Biol 80: 509–515. 12440692 10.1139/o02-142

[46] Smythe E, Ayscough KR (2006) Actin regulation in endocytosis. J Cell Sci 119: 4589–4598. 17093263 10.1242/jcs.03247

[47] Cox D, Greenberg S (2001) Phagocytic signaling strategies: Fc(gamma) receptor-mediated phagocytosis as a model system. Semin Immunol 13: 339–345. 11708889 10.1006/smim.2001.0330

[48] Swanson JA, Johnson MT, Beningo K, Post P, Mooseker M, et al (1999) A contractile activity that closes phagosomes in macrophages. J Cell Sci 112 (Pt 3) 307–316. 9885284 10.1242/jcs.112.3.307

[49] Jonsdottir GA, Li R (2004) Dynamics of yeast Myosin I: evidence for a possible role in scission of endocytic vesicles. Curr Biol 14: 1604–1609. 15341750 10.1016/j.cub.2004.08.055

[50] Ostap EM, Maupin P, Doberstein SK, Baines IC, Korn ED, et al (2003) Dynamic localization of myosin-I to endocytic structures in *Acanthamoeba* . Cell Motil Cytoskeleton 54: 29–40. 12451593 10.1002/cm.10081

[51] Reymann AC, Boujemaa-Paterski R, Martiel JL, Guerin C, Cao W, et al (2012) Actin network architecture can determine myosin motor activity. Science 336: 1310–1314. 22679097 10.1126/science.1221708 PMC3649007

[52] Diamond LS, Harlow DR, Cunnick CC (1978) A new medium for the axenic cultivation of Entamoeba histolytica and other *Entamoeba* . Trans R Soc Trop Med Hyg 72: 431–432. 212851 10.1016/0035-9203(78)90144-x

[53] Cormack BP, Valdivia RH, Falkow S (1996) FACS-optimized mutants of the green fluorescent protein (GFP). Gene 173: 33–38. 8707053 10.1016/0378-1119(95)00685-0

[54] Hamann L, Nickel R, Tannich E (1995) Transfection and continuous expression of heterologous genes in the protozoan parasite *Entamoeba histolytica* . Proc Natl Acad Sci U S A 92: 8975–8979. 7568055 10.1073/pnas.92.19.8975 PMC41090

[55] Hamann L, Buss H, Tannich E (1997) Tetracycline-controlled gene expression in *Entamoeba histolytica* . Mol Biochem Parasitol 84: 83–91. 9041523 10.1016/s0166-6851(96)02771-5

[56] Voigt H, Olivo JC, Sansonetti P, Guillen N (1999) Myosin IB from *Entamoeba histolytica* is involved in phagocytosis of human erythrocytes. J Cell Sci 112 (Pt 8) 1191–1201. 10085254 10.1242/jcs.112.8.1191

[57] Charuk JH, Pirraglia CA, Reithmeier RA (1990) Interaction of ruthenium red with Ca2(+)-binding proteins. Anal Biochem 188: 123–131. 1699445 10.1016/0003-2697(90)90539-l

