# Supplementary figures and images for: The Calmodulin-like Calcium Binding Protein EhCaBP3 of Entamoeba histolytica Regulates Phagocytosis and Is Involved in Actin Dynamics
Source: PLoS Pathog. 2012 Dec 27;8(12):e1003055. doi: 10.1371/journal.ppat.1003055 (PMC3531509; doi:10.1371/journal.ppat.1003055)

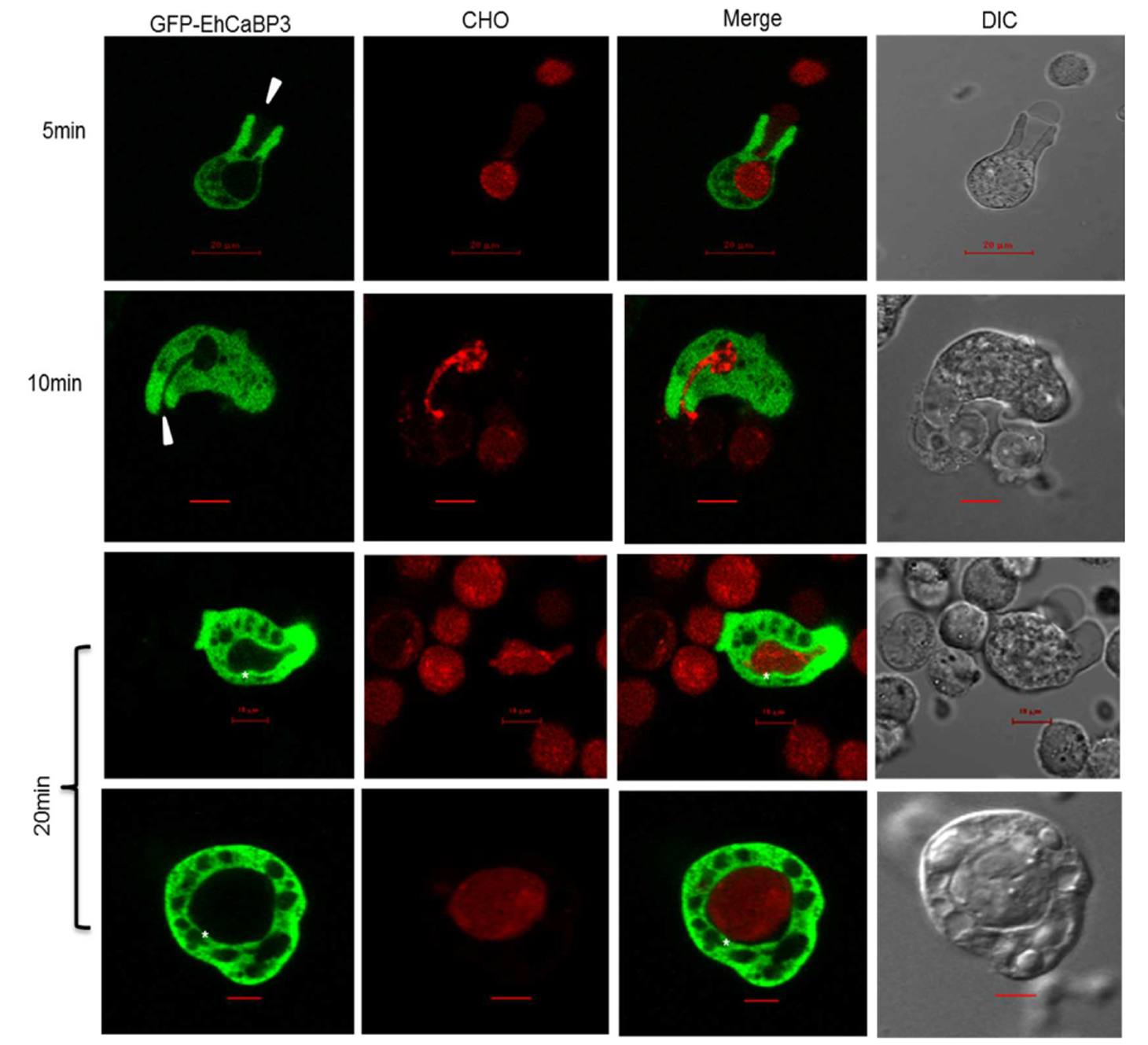

Supplement: Figure S1 — Localization of GFP–EhCaBP3 during CHO cell phagocytosis. CHO cells were labelled with Cell tracker orange and added to the cells expressing GFP–EhCaBP3 (approximately 1∶2 ratio). Cells were fixed and labelled with anti-GFP antibody, followed by Alexa-mouse 488. Arrows depict the phagocytic cups and asterisk indicates phagosomes containing CHO cells. Scale bar represents 20 µm. (PPT) [file ppat.1003055.s001.ppt]

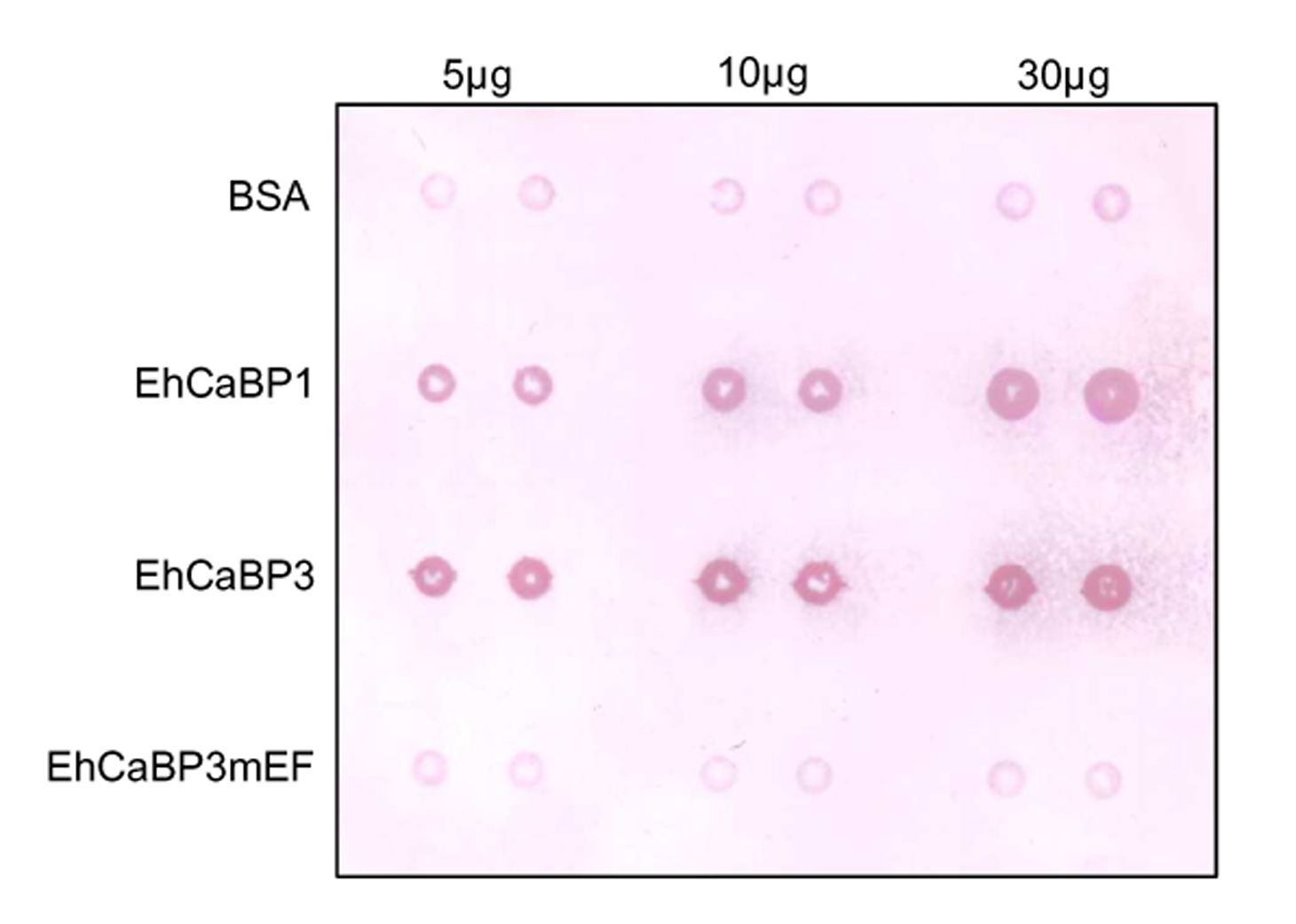

Supplement: Figure S2 — Calcium ability test by Ruthenium red staining. Purified recombinant proteins were blotted onto PVDF membrane, followed by washing. Then 25 µg ml−1 ruthenium red stain was added. EhCaBP1 and BSA were used as positive and negative controls respectively. (PPT) [file ppat.1003055.s002.ppt]

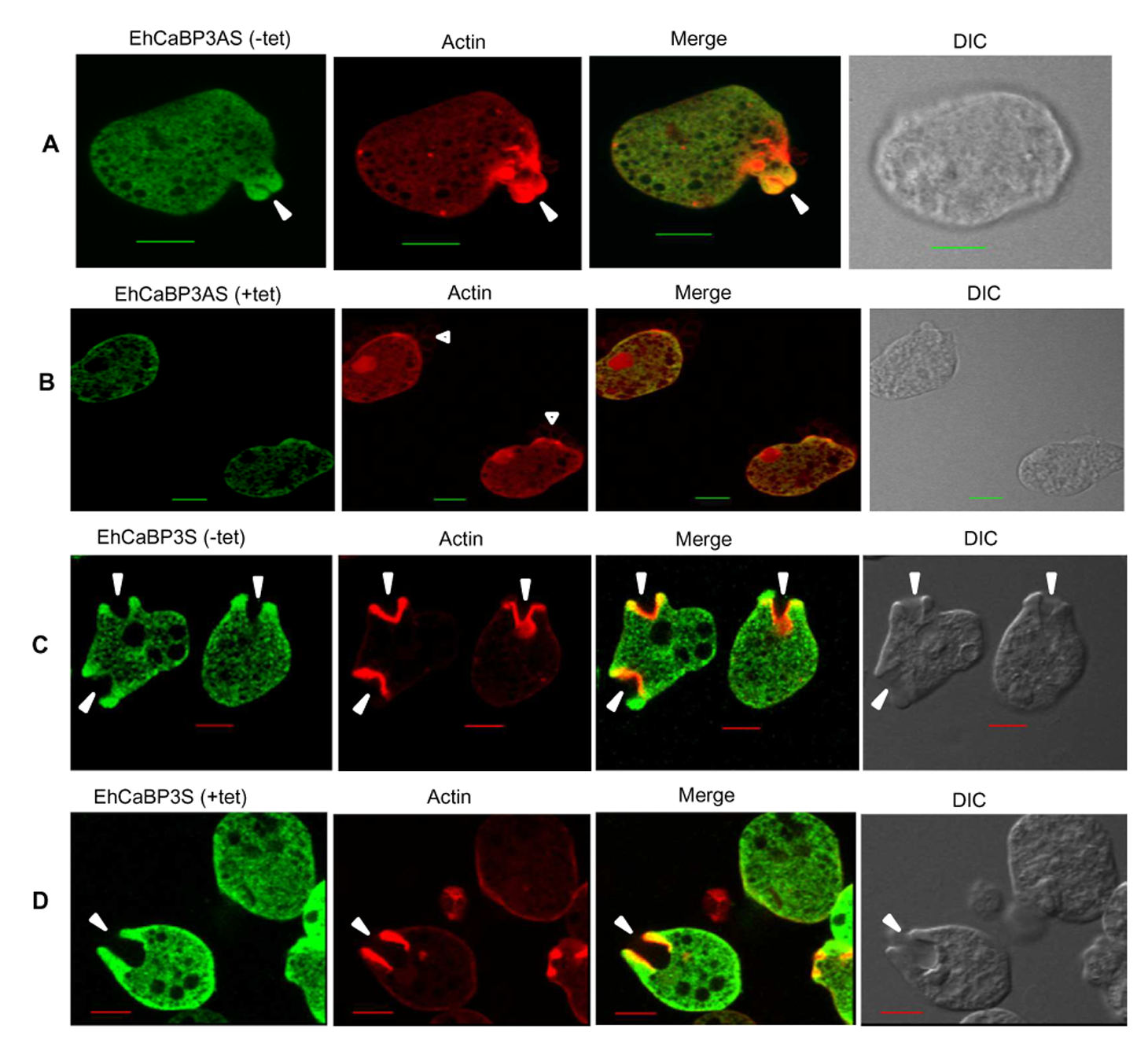

Supplement: Figure S3 — Cells expressing the anti-sense (A–B) and the sense (C–D) constructs in presence and absence of tetracycline were incubated with RBCs for 7 min. The cells were then fixed and stained with Alexa 488 (EhCaBP3) and TRITC-phalloidin (actin). Solid arrow heads represent the phagocytic cups, open arrow shows attached RBC. (Scale bar, 10 µm; DIC, differential interference contrast). (PPT) [file ppat.1003055.s003.ppt]

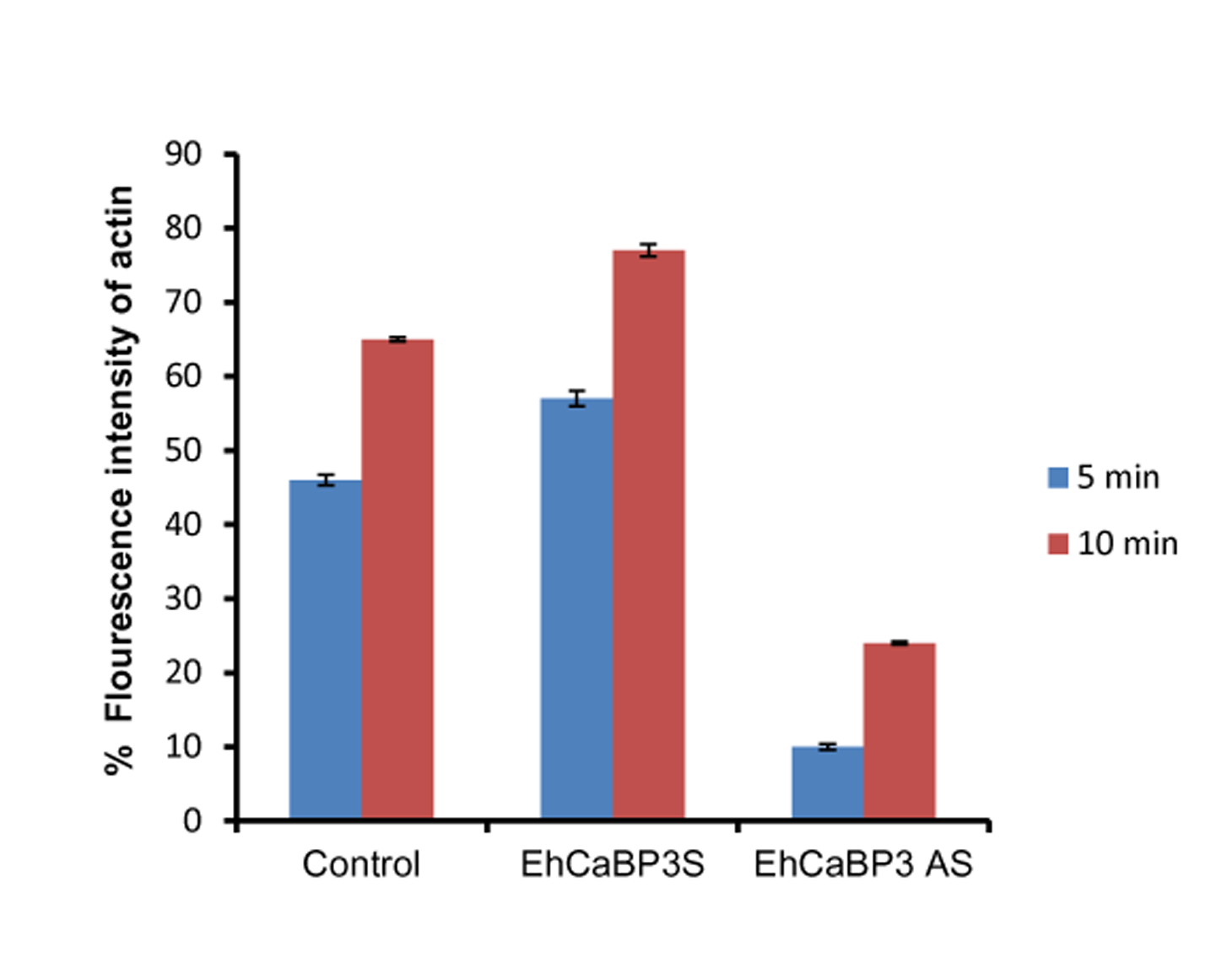

Supplement: Figure S4 — Quantitation of phalloidin staining in anti-sense phagocytosing cells. Intensity of F-actin was measured at multiple locations in the cytosol and in phagocytic cups. Average relative intensity of phagocytic cups was computed by taking the signal from cytosol as 100%. (N = 10, bars represent standard error). (PPT) [file ppat.1003055.s004.ppt]

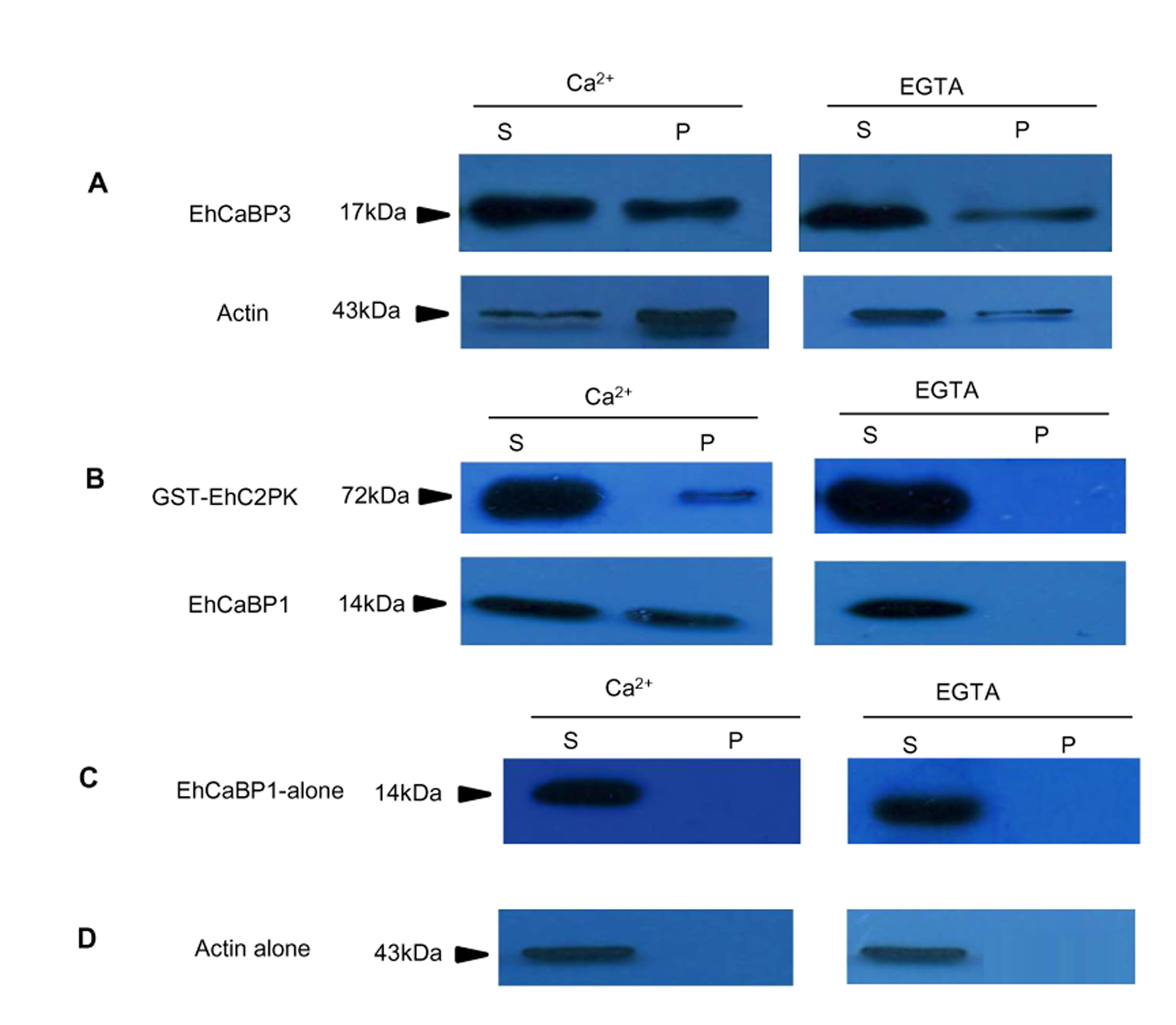

Supplement: Figure S5 — Binding of EhCaBP3 to phosphatidylserine. Presence of specific proteins in the pellet (P), that is, liposome fraction indicates binding. Proteins that do not bind remain in the soluble fraction (S). (A) Purified EhCaBP3 and actin were incubated with phosphatidylserine (PS) liposomes in the presence and the absence of Ca2+. (B) Purified GST-EhC2PK and EhCaBP1 were incubated with liposomes in presence and absence of Ca2+. (C–D) EhCaBP1 and actin were incubated with liposomes. (PPT) [file ppat.1003055.s005.ppt]
