# Supplementary material for: The Calmodulin-like Calcium Binding Protein EhCaBP3 of Entamoeba histolytica Regulates Phagocytosis and Is Involved in Actin Dynamics
Source: PLoS Pathog. 2012 Dec 27;8(12):e1003055. doi: 10.1371/journal.ppat.1003055 (PMC3531509; doi:10.1371/journal.ppat.1003055)
Supplement: Table S1 — List of oligonucleotides used in the study. (DOC) [file ppat.1003055.s007.doc]

| **NAME OF THE OLIGOS**  **Supplementary Table S1.** List of oligonucleotides used in the study. | | **SEQUENCE** |
| --- | --- | --- |
| EhCaBP3 Sense(S) Fp | | 5´-CGGGGTACCATGAGTGAACAAAAGAAGGTA-3´ |
| EhCaBP3 Sense (S) Rp | | 5´-CGCGGATCCTTATTTGCTGCTAATCAATTTAAC-3´ |
| EhCaBP3 Anti-Sense (AS) Fp | | 5´-CGCGGATCCATGAGTGAACAAAAGAAGGTA-3´ |
| EhCaBP3 Anti-sense (AS) Rp | | 5´-CGGGGTACCTTATTTGCTGCTAATCAATTTAAC-3 |
| EhCaBP3- GFP Fp | | 5´-CGCGGATCCATGAGTGAACAAAAGAAGGTA-3´ |
| EhCaBP3-GFP Rp | | 5´-CGCGGATCCTTATTTGCTGCTAATCAATTTAAC-3´ |
| **PRIMERS FOR SITE DIRECTED MUTAGENESIS** | | |
| **OLIGOS** | | **MUTATION INCORPORATED** |
| EhCaBP3 E35-A Fp and EhCaBP3 E35-A Rp | | Glutamate at position 35 mutated to alanine (A) |
| EhCaBP3 E108-A Fp and EhCaBP3 E108-A Rp | | Glutamate at position 108 mutated to alanine (A) |
| EhCaBP3 D24-A Fp and EhCaBP3 D24-A Rp | | Aspartate at position 24 mutated to alanine (A) |
| EhCaBP3 D60-A Fp and EhCaBP3 D60-A Rp | | Aspartate at position 60 mutated to alanine (A) |
| EhCaBP3 D97-A Fp and EhCaBP3 D97-A Rp | | Aspartate at position 97 mutated to alanine (A) |
| **SEQUENCES OF OLIGOS USED FOR SITE DIRECTED MUTAGENESIS** | | |
| **OLIGOS** | **SEQUENCE** | |
| EhCaBP3 E35-A Fp | 5’GCTTACAGCTGAAGCACTCTGAACAGTTA 3’ | |
| EhCaBP3 E35-A Rp | 5’TAACTGTTCCGAGTGCTTCAGCTGTAAGC 3’ | |
| EhCaBP3 E108-A Fp | 5’CTACATTTCAGCTTCAGCGCTTAAACATGTTTTAAC 3’ | |
| EhCaBP3 E108-A Rp | 5’GTTAAAACATGTTTAAGCGCTGAAGCTGAAATGTAG 3’ | |
| EhCaBP3 D24-A Fp | 5’ TTCCAATTGTTTGCTAAGGATAATGAT 3’ | |
| EhCaBP3 D24-A Rp | 5’ATCATTATCCTTAGCAAACAATTGGAA 3’ | |
| EhCaBP3 D60-A Fp | 5’ GTCAAAGATTATGCTAAAGATAATAGTG 3’ | |
| EhCaBP3 D60-A Rp | 5’ CACTATTATCTTTAGCATAATCTTTGAC 3’ | |
| EhCaBP3 D97-A Fp | 5’ CATTTGAAATTTTTGCTAAAGAAAAGAATG 3’ | |
| EhCaBP3 D97-A Rp | 5’ CATTCTTTTCTTTAGCAAAAATTTCAAATG 3’ | |
