# Supplementary material for: The Calmodulin-like Calcium Binding Protein EhCaBP3 of Entamoeba histolytica Regulates Phagocytosis and Is Involved in Actin Dynamics
Source: PLoS Pathog. 2012 Dec 27;8(12):e1003055. doi: 10.1371/journal.ppat.1003055 (PMC3531509; doi:10.1371/journal.ppat.1003055)
Supplement: Text S1 — Supplementary methods. (DOC) [file ppat.1003055.s008.doc]

**Experimental Procedures**

***Actin bundling assay***

Muscle G-actin (Sigma) 3 µM per reaction was polymerized for 1 hour in polymerization buffer containing 100 mM KCl and 2 mM MgCl_2_ at room temperature. The purified proteins (5 µM), precentrifuged to remove aggregates were added in a total volume of 100 µl of buffer (10 mM Tris-HCl, pH 7.5,1 mM CaCl_2_, 2.5 mM β-mercaptoethanol, 0.5 M KCl, 10 mM MgCl_2_, 0.2 mM ATP) and incubated for 2 hours at room temperature. The samples were then centrifuged at 10,000 × g for 20min to sediment bundled F-actin. The pellet and supernatant were subjected to SDS-PAGE, followed by Coomassie brilliant blue (CBB) staining. In addition to WT (CaBP3), EhCaBP1 and alpha-actinin were also used as positive controls while BSA was used as a negative control.

***Erythrophagocytosis assay***

Erythrophagocytosis assay was carried out as previously described [21]. Briefly, RBCs (10^7^ ) were first washed with PBS and incomplete TYI-33 medium and then incubated with (10^5^) amoebae for indicated time points at 37°C in 0.5 ml of culture medium. Amoebae and RBCs were pelleted down and non-engulfed RBCs were lysed with cold distilled water and centrifuged at 1000*g* for 2 min. This step was repeated twice, followed by re-suspension in 1 ml formic acid to burst amoebae containing engulfed RBC. The absorbance was measured by a spectrophotometer at 400 nm using formic acid as blank.

***Ruthenium red staining***

Ruthenium red staining was done as described by Charuk et al., 1990 [57]. Briefly, different amounts of purified recombinant proteins were first blotted (in duplicates) on to a PVDF membrane (Millipore). The membrane was washed thrice for 20min with a washing buffer (10mM Imidazole HCl, 60mM KCl and 5mM MgCl_2_ ,pH 6.8) and then incubated in the same buffer containing 25 μg/ml ruthenium red (Sigma-aldrich, U.S.A) overnight. The membrane was subsequently rinsed for 2 min in distilled chelex treated water and then stored in distilled water. In this assay, EhCaBP1 and BSA were used as positive and negative controls respectively.
